# Catalytic Fast Pyrolysis of Kraft Lignin With Conventional, Mesoporous and Nanosized ZSM-5 Zeolite for the Production of Alkyl-Phenols and Aromatics

**DOI:** 10.3389/fchem.2018.00295

**Published:** 2018-07-18

**Authors:** Polykarpos A. Lazaridis, Apostolos P. Fotopoulos, Stamatia A. Karakoulia, Konstantinos S. Triantafyllidis

**Affiliations:** ^1^Department of Chemistry, Aristotle University of Thessaloniki, Thessaloniki, Greece; ^2^Chemical Process and Energy Resources Institute, Centre for Research and Technology Hellas, Thessaloniki, Greece

**Keywords:** kraft softwood lignin, fast pyrolysis, bio-oil, ZSM-5 zeolite, mesoporosity, alkyl-phenols, aromatics, hierarchical MFI

## Abstract

The valorization of lignin that derives as by product in various biomass conversion processes has become a major research and technological objective. The potential of the production of valuable mono-aromatics (BTX and others) and (alkyl)phenols by catalytic fast pyrolysis of lignin is investigated in this work by the use of ZSM-5 zeolites with different acidic and porosity characteristics. More specifically, conventional microporous ZSM-5 (Si/Al = 11.5, 25, 40), nano-sized (≤20 nm, by direct synthesis) and mesoporous (9 nm, by mild alkaline treatment) ZSM-5 zeolites were tested in the fast pyrolysis of a softwood kraft lignin at 400–600°C on a Py/GC-MS system and a fixed-bed reactor unit. The composition of lignin (FT-IR, 2D HSQC NMR) was correlated with the composition of the thermal (non-catalytic) pyrolysis oil, while the effect of pyrolysis temperature and catalyst-to-lignin (C/L) ratio, as well as of the Si/Al ratio, acidity, micro/mesoporosity and nano-size of ZSM-5, on bio-oil composition was thoroughly investigated. It was shown that the conventional microporous ZSM-5 zeolites are more selective toward mono-aromatics while the nano-sized and mesoporous ZSM-5 exhibited also high selectivity for (alkyl)phenols. However, the nano-sized ZSM-5 zeolite exhibited the lowest yield of organic bio-oil and highest production of water, coke and non-condensable gases compared to the conventional microporous and mesoporous ZSM-5 zeolites.

## Introduction

Lignocellulosic biomass is considered as an alternative source of fuels/energy, chemicals and products, with the potential to replace, at least partially, the fossil raw materials, i.e., petroleum oil and coal (Huber et al., 2006; Triantafyllidis, 2013). Biomass consists mainly of two carbohydrate polymers (polysaccharides), i.e., cellulose (30–50%) and hemicellulose (15–30%) and the phenolic polymer lignin (10–30%) (Mosier et al., 2005; Azadi et al., 2013; Isikgor and Becer, 2015). Lignin is nature's most abundant aromatic and water insoluble polymer, consisting of three primary phenylpropane units, i.e., syringyl (S), guaiacyl (G), and p-hydroxyphenyl (H) units which correspond to three monolignols, i.e., sinapyl, coniferyl and p-coumaryl alcohols, all three joined by ether and C–C linkages to form the 3-dimensional macromolecule of lignin (Zakzeski et al., 2010; Azadi et al., 2013; Li et al., 2015; Rinaldi et al., 2016). The most common linkages in lignin structure are ether linkages, such as the β-O-4 which predominates (40–50%) and α-O-4, as well as the 5-5, β-5, β-1, dibenzodioxocin, spirodienone and β-β linkages (Chakar and Ragauskas, 2004; Zakzeski et al., 2010; Rinaldi et al., 2016). The content of lignin depends on the type of raw biomass, i.e., 20–30 wt.% in softwoods (i.e., spruce, pine, fir, yew), 15–25 wt.% in hardwoods (i.e., beech, aspen, birch, oak, poplar) and 10–20 wt.% in grasses, straw and stover. The structure and composition of lignin is also related to the type of plant species, i.e., softwood lignins contain mainly guaiacyl (coniferyl alcohol) units with one methoxy group attached to the aromatic ring, hardwood lignins contain both guaiacyl and syringyl (sinapyl alcohol) units, the latter having two methoxy groups, while grass lignins contain also p-hydroxyphenyl (p-coumaryl alcohol) units in addition to the G- and S-units (Azadi et al., 2013; Li et al., 2015).

In traditional wood valorization industry (i.e., pulping/Kraft process) or more recently developed biorefining processes, lignin has been considered as a low-value byproduct that can be burnt to generate heat and power. However, over the last two decades, this low-cost raw material has found increased valorization potential in various sectors, such as polymers/resins, binders, foams, cement, carbon fibers and activated carbons, vanillin, etc. (Setua et al., 2000; Lora and Glasser, 2002; Suhas et al., 2007; Silva et al., 2009; Pandey and Kim, 2011; Strassberger et al., 2014). Nowadays, intensive research is being conducted on the development of efficient processes for the thermo-catalytic and bio-catalytic conversion of this aromatic/phenolic natural polymer toward high value chemicals and fuels (Ragauskas et al., 2014; Xu et al., 2014; Li et al., 2015; Beckham et al., 2016). Most studied depolymerization-valorization processes of kraft and other types of lignin (i.e., hydrolysis, organosolv) are hydrogenolysis and fast pyrolysis. The hydrogenolysis of lignin usually requires moderate reaction temperature (ca. 150–350°C), high hydrogen pressure (ca. 20–90 bar) and/or hydrogen-donor solvents (catalytic transfer hydrogenation) in neutral/acidic/basic medium, using supported metal catalysts such as Pt, Ni, Pd, Cr, Cu etc. on carbon, carbides, zeolites and various oxides, targeting to cleavage of the C-O bonds (ca. β-O-4 ether bond) and C-C bonds in order to produce low molecular weight fragments or monomeric phenolic/aromatic/alkanes compounds (Zakzeski et al., 2010; Sergeev and Hartwig, 2011; Xu et al., 2012; Barta et al., 2014; Onwudili and Williams, 2014; Liu et al., 2016; Molinari et al., 2016; Opris et al., 2017).

Fast pyrolysis is a relatively intense, in terms of temperature (400–700°C), thermochemical process which is capable to break-down lignin into smaller fragments in the absence of oxygen, toward the production of bio-oil which consists mainly of alkoxy-phenols and oxygenated aromatics (e.g., guaiacol, methyl guaiacol, syringol, methyl syringol, vanillin, syringaldehyde, vinyl syringol, vinyl guaiacol, 1,2,3-trimethoxy-benzene), as well as some gaseous products (mainly CO_2_ and CO) and char (Fox and McDonald, 2010; Jiang et al., 2010; Pandey and Kim, 2011; Patwardhan et al., 2011; Li et al., 2015). The lignin-derived pyrolysis oil is more homogeneous compared to the bio-oil that derives from the parent lignocellulosic biomass, the latter consisting, in addition to the phenolic compounds, of various ketones, aldehydes, acids, furans, esters, ethers, alcohols, sugars and few aromatics and aliphatics (Azeez et al., 2010; Stephanidis et al., 2011). Such a phenolic bio-oil, derived from lignin, has greater potential of being utilized in the production of phenol-based resins substituting the petroleum derived phenol (Vithanage et al., 2017). Alternatively, the lignin-derived bio-oil can be upgraded via down-stream catalytic hydrodeoxygenation (HDO) to produce hydrocarbons, mainly aromatics and (cyclo)alkanes. The HDO process requires moderate reaction temperatures (ca. 150–300°C), ambient to high hydrogen pressures (ca. up to 50 bars) and/or hydrogen-donor solvents in neutral/acidic medium in the case of liquid phase processing, using various supported catalytic systems, such as typical petroleum hytrotreating catalysts, i.e., bimetallic catalyst sulfided CoMo, NiMo/γ-Al_2_O_3_ (Laurent and Delmon, 1994; Ryymin et al., 2010), unsupported and alumina-supported MoS_2_ and CoMoS catalysts (Bui et al., 2011), Mo_2_C catalysts (Lee et al., 2014), combined systems of a hydrogenating and an acidic catalyst, i.e., Pd/C and H-ZSM-5 (Zhao and Lercher, 2012), and various Ni, Pd, Pt, Ru, etc. catalysts supported on carbon, SiO_2_, Al_2_O_3_, TiO_2_, ZrO_2_, CeO_2_, zeolites, etc. (Mortensen et al., 2013; Jin et al., 2014; de Souza et al., 2017; Kordouli et al., 2017).

The bio-oil can also be *in situ* deoxygenated, i.e., during the biomass fast pyrolysis process by the use of an appropriate, usually acidic catalyst. In the catalytic fast pyrolysis (CFP) of biomass the initially formed (via thermal pyrolysis) vapors of oxygenated oligomers and monomers (i.e., ketones, aldehydes, furans, acids, alkoxy-phenols, etc.) undergo deoxygenation (dehydration, decarbonylation, decarboxylation), cracking, isomerization, aromatization, condensation and oligomerization reactions on the catalyst surface. The use of catalysts with strong Brønsted acidity, such as zeolites and especially ZSM-5 zeolite, induce deep-deoxygenation of the produced bio-oil which consist mainly of mono-aromatics (i.e., benzene, toluene, xylenes, etc.) and naphthalenes, as well as alkyl-phenols. Inevitably, more water, gases and coke-on-catalyst are being produced at the expense of the organic phase of bio-oil (Iliopoulou et al., 2007, 2017; Jae et al., 2011; Mihalcik et al., 2011; Wang et al., 2014; Thommes et al., 2015). In the case of lignin CFP, the same concept, catalysts and reaction mechanisms apply (Mullen and Boateng, 2010; Ma et al., 2012; Ben and Ragauskas, 2013). Most studies have investigated the performance of different microporous zeolitic catalysts (i.e., ZSM-5 with various Si/Al ratio, Beta, Mordenite, Ferrierite, USY) (Jackson et al., 2009; Mihalcik et al., 2011; Li et al., 2012; Ma et al., 2012; Ben and Ragauskas, 2013; Zhang et al., 2014). As with biomass CFP, H-ZSM-5 zeolite has been identified as the most suitable for the production of aromatics, due to its unique micropore structure and Brønsted acidity strength. Still, USY zeolite with larger micropores than those of ZSM-5, has been also proposed as very efficient for the production of hydrocarbons in the CFP of kraft lignin, exhibiting also relatively lower degree of tar formation (Ma et al., 2012). More recently, the use of high surface area ordered mesoporous Al- or Zr-substituted silicas, with average pore width in the range of ca. 3–10 nm, has been also investigated (Custodis et al., 2016; Elfadly et al., 2016). Custodis et al. studied the effect of acidity and porosity of Al-MCM-41, Al-SBA-15, and Al-MSU-J on product yields and bio-oil composition in the CFP of alkali softwood lignin, showing that a nano-sized Al-MCM-41 catalyst may exhibit similar deoxygenation and aromatization activity as a strongly acidic microporous ZSM-5 zeolite (Custodis et al., 2016).

The development of mesoporous zeolites or “hierarchical” zeolites (i.e., comprising of micro-, meso- and macro-porous structures) has attracted the interest for the cracking/pyrolysis of heavy oil fractions (Choi et al., 2006; Park et al., 2009) as well as lignocellulosic biomass (Park et al., 2010; Kelkar et al., 2014; Li et al., 2014; Zheng et al., 2014; Gamliel et al., 2016), aiming to combine the beneficial diffusion characteristics of mesoporous materials with the strong acidity and stability of zeolites. Mesoporous MFI (ZSM-5) zeolites, having in some cases moderate/tuned acidity or even acid-base properties (Kelkar et al., 2014; Fermoso et al., 2016), have been the main type of hierarchical zeolites studied for biomass pyrolysis. With regard to lignin CFP, very few studies have been reported yet on the use hierarchical zeolites. Lee et al. tested a meso-Y zeolite (prepared from commercial USY) in the CFP of kraft lignin and showed that the production of mono-aromatics and polycyclic aromatic hydrocarbons (PAHs) was significantly enhanced compared to Al-MCM-41 which produced mostly phenolics (Lee et al., 2013). In addition Kim et al. showed that the main products in lignin pyrolysis with mesoporous MFI zeolite were alkyl-phenols and mono-aromatics (benzene, toluene, ethylbenzene, and xylene) owing to the strong Brønsted acidity of the zeolite in comparison to mesoporous Al-SBA-15 (Kim et al., 2014). Li et al. also showed that in the CFP of lignin by alkaline-treated mesoporous ZSM-5 zeolite the aromatics and phenols were increased while char/coke was decreased compared to the parent microporous zeolite (Li et al., 2014).

In the present work, we studied the catalytic fast pyrolysis of kraft lignin (spruce, softwood) using a series of conventional microporous ZSM-5 zeolites (with different Si/Al ratio), a nanosized crystalline ZSM-5 with high textural porosity/external area and a mesoporous ZSM-5 zeolite with intracrystal mesoporosity prepared by mild alkaline treatment of a commercial ZSM-5 zeolite. The non-catalytic and catalytic tests of lignin pyrolysis were conducted on a Pyrolyzer/Gas Chromatography-Mass Spectrometry (Py/GC-MS) instrument at different temperatures (400–600°C) and catalyst to lignin ratios (1–4), in order to study the effect of the various ZSM-5 catalysts on the composition of bio-oil. Furthermore, a fixed bed fast pyrolysis unit was also used, in order to determine the product yields (bio-oil, gases, char/coke). The catalytic resutls have been reationalized on the basis of the catalysts' acidic, porous and morphology characterisics and reaction mechanisms for the deoxygenation of the initially formed alkoxy-phenols toward alkyl-phenols and aromatics have been proposed.

## Materials and methods

### Catalyst preparation

Three commercial ZSM-5 zeolites with different Si/Al ratio (provided by Zeolyst) were tested: CBV 2314 (Si/Al = 11.5), CBV 5524G (Si/Al = 25), CBV 8014 (Si/Al = 40). All the commercial samples were received in ammonium form and were converted to proton form via calcination at 500°C for 3 h in air flow prior to use and were denoted as ZSM-5 (11.5), ZSM-5 (25), and ZSM-5 (40) respectively. A mesoporous ZSM-5 sample, denoted as Meso-ZSM-5 (9nm), was prepared by mild alkaline treatment of the commercial H-CBV8014 (Si/Al = 40) zeolite with 0.2 M NaOH aq. solution, followed by treatment with 0.1 N HCl aq. solution (the detailed procedure is described in Supplementary Material). The nanosized zeolite (Nano-ZSM-5) was synthesized based on typical template hydrothermal methods applied for ZSM-5 zeolite, using tetraethylorthosilicate (TEOS, 98%, Sigma-Aldrich) and aluminum-tri-sec-butoxide (97%, Sigma-Aldrich) as Si and Al sources, and 1.0 M-Tetrapropylammonium hydroxide (TPAOH, Sigma-Aldrich) solution as the structure-directing agent for MFI-type zeolite. In brief, TEOS and Al-tri-sec-butoxide were initially mixed under stirring followed by addition of the TPAOH solution (mixture molar ratio: 1 SiO_2_: 0.01 Al_2_O_3_: 0.37 TPAOH: 16.4 H_2_O) and further stirring for 1 h at room temperature. The hydrothermal aging of the resulting suspension was conducted at 100°C for 4 days, followed by filtration of the resulting solids, washing (with plenty of water), drying (100°C overnight) and calcination (600°C, 6 h, in air). All zeolite powders were pelletized, crushed and sieved to a particle size range of 180–500μm before use in the fixed bed lignin pyrolysis experiments.

### Characterization of kraft lignin

The kraft lignin (from spruce, softwood) used in this study was provided by Sigma-Aldrich. The elemental analysis of dried kraft lignin sample was performed by a Carbon/Hydrogen/Nitrogen/Sulfur elemental analyzer (LECO 628 and LECO 932, USA). Oxygen content was calculated by difference. Thermogravimetric analysis (TGA, NETZSCH STA 449 F5 Jupiter) of the dried kraft lignin sample was carried out using N_2_ as carrier gas (purity >99.99 vol %) at a flow rate of 50 mL/min. The samples were heated from room temperature to 850°C at heating rate of 10°C/min. FTIR spectra were obtained on a Perkin-Elmer FTIR spectrometer, model SPECTRUM 1000, using the KBr method (KBr was previously oven-dried to avoid interferences due to the presence of water). Measurements were carried out using thin disks prepared in a hydraulic press and spectra recorded over the range from 4,000 to 400 cm^−1^ at a resolution of 2 cm^−1^ while 64 scans were averaged to reduce noise. The spectra presented were baseline-corrected and converted to the absorbance mode.

The molecular weight distribution (MWD) and the average molecular weight of kraft lignin were determined by Gel Permeation Chromatography (GPC). The instrument used was from Polymer Laboratories, model PL-GPC 50 Plus, and comprised of an isocratic pump, a differential refractive index detector, and three PLgel 5l MIXED-C columns in series. Approximately 5 mg of lignin were suspended in 1 mL glacial acetic acid/acetyl bromide (9:1 v/v) for ~2 h (Asikkala et al., 2012). Afterwards the solvent was fully removed in vacuum evaporator, and the residue sample was dissolved in THF at a constant concentration of 1 mg/mL and sonicated overnight. After filtration with PTFE-L 0.45 μm, 200 μL of sample was injected into the chromatograph. The elution solvent was THF (HPLC grade) at a constant flow rate of 1 mL/min, and the entire system was kept at a constant temperature of 30°C. Calibration of GPC was carried out with standard polystyrene samples (Polymer Laboratories).

The 2D HSQC NMR spectrum was obtained on a Varian (Agilent) 500 MHz spectrometer. 200 mg of Kraft lignin were dissolved in 0.45 ml DMSO-d6 and stirred overnight before the analysis and chemical shifts were referenced to the solvent signal (2.500/39.520 ppm). The interscan relaxation delay was set to 5 s that has been found to be sufficient time for full relaxation of the signals (Heikkinen et al., 2003). The spectral widths were from 13 to −1 ppm and from 160 to 0 ppm for the ^1^H and ^13^C dimensions, respectively. In the ^13^C dimension the number of transients was 16 and the increments were set at 300. The spectrum was processed using MestReNova software. Prior to Fourier transformation, FIDs were apodized with a π/2 sine square bell function in both dimensions and zero-filled up to 1,024 points in the ^13^C dimension and 2,048 points in the ^1^H-dimension. A semi-quantitative analysis of the heteronuclear single quantum coherence (HSQC) spectra was performed by integration of cross-peaks in the different regions of the spectra with MestReNova. In the HSQC spectrum, the signal of the G2 (C2-H) aromatic units was used as internal standard. More specifically, the area obtained from the integration of the G2 unit was set as 100 aromatic units and the amount of each linkage type (expressed as a number per 100 Ar) was calculated by the equation:

$$X\;=\;\frac{\int \;X}{\int \;G2}\;\times \;100$$

### Characterization of catalytic materials

The chemical composition (wt. % of Al and Na) of the zeolitic catalysts was determined by inductive coupled plasma—atomic emission spectroscopy (ICP-AES) using a Plasma 400 (Perkin Elmer) spectrometer, equipped with Cetac6000AT+ ultrasonic nebulizer.

Nitrogen adsorption/desorption experiments at −196°C were performed on an Automatic Volumetric Sorption Analyzer (Autosorb-1MP, Quantachrome). The samples were previously outgassed at 350°C for 16 h under 5 × 10^−9^ Torr vacuum. The BET area (i.e., total surface area) of the catalysts was determined by the multi-point BET method, the mesopore width distribution by the BJH analysis of the adsorption data, and the micropore volume and area by the t-plot method. Argon (Ar) physisorption measurements at −186°C were also performed, using the multi-point BET method for total surface area determination and the t-plot method for determination of the microporous characteristics. The NLDFT adsorption model was applied for the pore width distribution analysis. The BJH analysis was also used in the mesopore range for comparison.

Transmission electron microscopy (TEM and HRTEM) experiments were carried out in a JEOL 2011 high resolution transmission electron microscope operating at 200 kV, with a point resolution of 0.23 nm and Cs = 1.0 mm.

Powder X-ray diffraction (XRD) was applied for the determination of the crystallinity of zeolites using a Rigaku Rotaflex 200B diffractometer equipped with Cu Kα X-ray radiation and a curved crystal graphite monochromator operating at 45 kV and 100 mA; counts were accumulated in the range of 5–75° 2θ every 0.02° (2θ) with counting time 2 sec per step.

The determination of the amount and relative strength of Brønsted and Lewis acid sites of the catalysts was performed by Fourier transform—infrared (FT-IR) spectroscopy combined with *in situ* adsorption of pyridine. The FT-IR spectra were recorded on a Nicolet 5700 FTIR spectrometer (resolution 4 cm^−1^) using the OMNIC software and a specially designed heated, high-vacuum IR cell with CaF_2_ windows. All samples were finely ground in a mortar and pressed in self-supported wafers (15mg/cm^2^). The wafers were outgassed *in situ* at 450°C for 1 h under high vacuum (10–6 mbar) and a background spectrum was recorded at 150°C. Adsorption/equilibration with pyridine vapors was then conducted at 150°C, by adding pulses of pyridine for 1 h at a total cell pressure of 1 mbar. Spectra were recorded at 150°C, after equilibration with pyridine at that temperature and after outgassing for 30 min at higher temperatures, i.e., 250, 350, and 450°C, in order to evaluate the strength of the acid sites. The bands at 1,545 cm^−1^ (pyridinium ions) and 1,450 cm^−1^ (coordinated pyridine) were used to identify and quantify the Brønsted and Lewis acid sites, respectively, by adopting the molar extinction coefficients provided by Emeis (Emeis, 1993).

### Pyrolysis experiments using Py/GC-MS system

The thermal and catalytic fast pyrolysis experiments of kraft lignin were performed on a Multi-Shot Micro-Pyrolyzer (EGA/PY-3030D, Frontier Laboratories, Japan) connected to a gas chromatographer – mass spectrometer system (GCMS-QP2010, Shimadzu). Detailed description of the experimental set-up and procedure is provided in Supplementary Material (Figure S1). In brief, for the thermal (non-catalytic) pyrolysis tests, a dried (80°C under vacuum for 6 h) mixture of 1 mg lignin and 2 mg silica sand (as inert heat carrier material) was loaded in a stainless steel cup which was instantaneously dropped in the hot reactor/furnace and pyrolysis was conducted at the preset temperatures of ca. 400, 500, and 600°C, for 12 s. In the catalytic fast pyrolysis (CFP) experiments, mixtures of 1 mg of dried lignin with 1–4mg of the various zeolites were used (catalyst to lignin ratio range: 1–4). Identification of mass spectra peaks was achieved by the use of the scientific library NIST11s. The derived compounds were classified and categorized in the following 16 groups-families: mono-aromatics (AR), aliphatics (ALI), phenols (PH), acids (AC), esters (EST), alcohols (AL), ethers (ETH), aldehydes (ALD), ketones (KET), polycyclic aromatic hydrocarbons (PAH's), sugars (SUG) nitrogen compounds (NIT), sulfur compounds (SUL), oxygenated aromatics (OxyAR), oxygenated phenols (OxyPH) and unidentified compounds (UN). At least three experiments were performed for each catalyst/condition and the reported data are the mean values with a standard deviation being below 10% in all cases.

### Pyrolysis experiments on fixed bed reactor

The thermal and catalytic pyrolysis tests were performed on a bench-scale fixed bed tubular reactor, made of stainless steel 316 and heated by a 3-zone furnace. A specially designed piston system was used to introduce lignin into the reactor. The amount of lignin (dried at 80°C under vacuum for 6 h) used in all experiments was 0.5 g and the amount of silica sand (thermal pyrolysis experiments, non-catalytic) or catalyst (in the catalytic experiments) was also 0.5 g. In a typical pyrolysis experiment, the solid lignin was inserted from the top of the reactor and was pushed down instantaneously with the aid of the piston in the hot reactor zone, where it was vaporized at 600°C. The produced pyrolysis vapors were then driven downwards through the catalyst's bed with the aid of a constant N_2_ flow (100cm^3^/min) for 20 min. The pyrolysis product vapors were condensed in pre-weighted spiral glass receivers placed in a cooling bath (dry ice). Afterwards, the obtained bio-oil was collected with absolute ethanol and analyzed by GC–MS (GCMS-QP2010, Shimadzu). For the identification of the produced bio-oil vapors, the NIST11s mass spectral library was used and the derived compounds were classified and categorized in the 16 groups-families, as in the case of Py/GC-MS experiments. The water content of bio-oil was determined by Karl-Fischer titration (ASTM E203-08), while the elemental analysis (C/H/N/S) of the organic fraction of the bio-oil was determined by LECO 628 and LECO 932 analyzers (USA); O was determined by difference.

The amount of solids, which comprised of char in the non-catalytic pyrolysis experiment and char plus coke-on-catalyst in the catalytic pyrolysis experiments, was determined by direct weighing. An indirect estimation of the coke formed on the catalyst, as wt.% on initial lignin, was performed by subtracting the measured char content of the non-catalytic experiment from the char+coke content of the catalytic experiments (char formation is not affected by the presence of the catalysts, as lignin and catalysts do not come in contact, see Supplementary Materials, Figure S2). Furthermore, the decomposition profile of the collected char and coke (on the spent catalysts) was studied by thermogravimetric analysis (TGA, NETZSCH STA 449 F5 Jupiter) using dry air as carrier gas, at a flow rate of 50mL/min. The samples were heated from room temperature to 850°C at heating rate of 10°C/min. Non-condensable gases (NGC's) were measured by the liquid displacement method and were analyzed by GC equipped with TCD and FID (HP5890 Series II). More details on the experimental set-up and analytic procedures are provided in Supplementary Material (Figure S2). The standard deviation of the product yield values reported is in all cases below 5%.

## Results and discussion

### Physicochemical characteristics of kraft lignin

The elemental analysis of the lignin used as feedstock is given in Table 1. The C/H/O content was typical for such type of lignins, while ~ 1.5% S was also measured owing to the kraft pulping process. The molecular weight of lignin was also in the range of previously reported values for similar lignins (Vishtal and Kraslawski, 2011; Li et al., 2012).

**Table 1:** Physicochemical characteristics of kraft lignin (dry basis).

**Lignin**	***C (wt.%)***	***H (wt.%)***	***S (wt.%)***	***N (wt.%)***	O^*^ (wt.%)	**Ash (wt.%)**	**Mn (g/mol)**	**Mw (g/mol)**	**PD**
Kraft (spruce)	61.84	5.62	1.50	0.80	30.24	2.04	1,350	6,140	4.54

* *Calculated by difference*.

**Figure 1:**
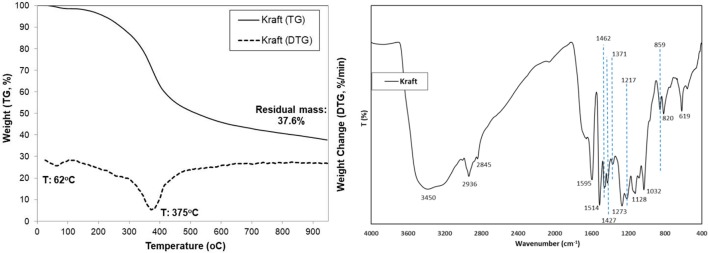
TGA and DTG curves (**left**) and FT-IR spectrum (**right**) of kraft lignin.

The thermal decomposition profile of lignin, both as TG (weight %) and DTG (%/min) curves, is shown in Figure 1. A small initial weight loss of 3–4% up to ca. 150°C was observed due to evaporation of humidity, followed by a steep decrease of weight (~55% loss, DTG peak maximum at 375°C) initiating at about 170–200°C and ending at about 600°C which corresponds to the decomposition of lignin. A further progressive limited decrease of weight was observed up to 900°C which is probably attributed to the slow pyrolysis/gasification of the initially formed carbonaceous material. The high residual char at 800°C (37.6% minus 2.04% ash: 35.5%) is indicative of the high thermal stability of the complex cross-linked aromatic polymer of lignin (Yang et al., 2006).

The FTIR spectrum of lignin is also presented in Figure 1. Assignment of representative peaks was based on previous reported work (Kang et al., 2012; Minu et al., 2012; Yang et al., 2016). A strong absorbance at 3,450 cm^−1^ assigned to -OH stretching vibrations was induced by the presence of alcoholic and phenolic hydroxyl groups involved in hydrogen bonds. Peaks at 2,936 and 2,845 cm^−1^ are assigned to C-H asymmetric and symmetrical vibrations of alkyls in side-chains (Zhang et al., 2012). Absorption bands located at around 1,595, 1,514, 1,462, and 1,427cm^−1^ were assigned to vibrations of aromatic rings, suggesting they were left intact and that the aromatic structure of lignin was not changed appreciably during the pulping process (Chen et al., 2016; Yang et al., 2016). The peaks at 1,217 and 1,273cm^−1^ were assigned to the vibrations of guaiacyl rings (Minu et al., 2012). The strong band at 1,032cm^−1^ corresponds to the aromatic C–H in-plain deformation (G>S units). The band at 859cm^−1^ represented the deformation vibrations of C-H bonds in the aromatic rings of p-hydroxyphenylpropane (Yang et al., 2016). The band at 820cm^−1^ is attributed to aromatic C–H out of plane vibrations. The absorption band at 620cm^−1^ indicate probably the presence of sulfonic groups (S-C) that remained after the kraft pulping process (Domínguez-Robles et al., 2016).

**Figure 2:**
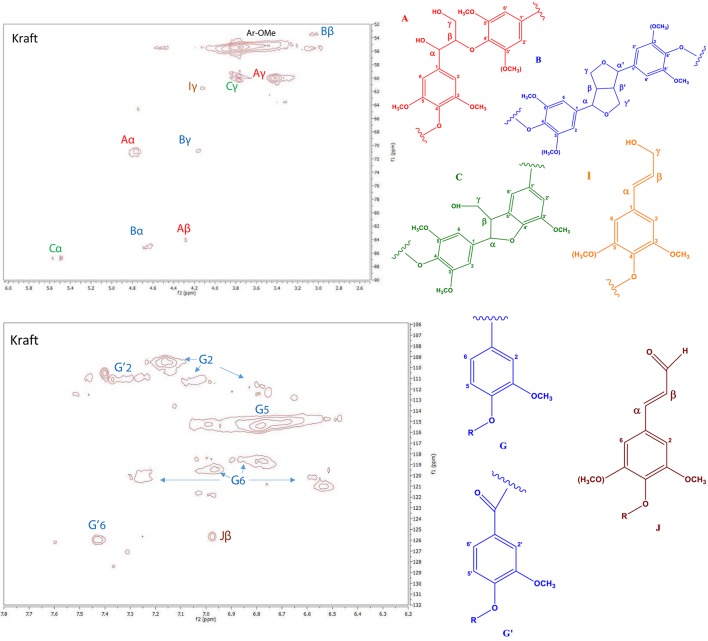
Side-chain (**top**) and aromatic regions (**bottom**) in the 2D HSQC NMR spectra of kraft lignin.

In order to study the structural characteristics of kraft lignin, the 2D HSQC NMR technique was applied. More specifically, the types of side-chain linkages and of aromatic units contained in the lignin were identified. The side-chain (δC/δH 50–95/2.5–6.0) and the aromatic (δC/δH 100–135/6–8) region of the HSQC spectrum of the kraft lignin sample is shown in Figure 2. Using literature data, the HSQC cross-peaks of the spectrum were assigned to specific types of aromatic rings and linkages (Table S1, Supplementary Material) (del Río et al., 2009; Ralph et al., 2009; Sette et al., 2011; Kang et al., 2012; Constant et al., 2016; Yang et al., 2016; Crestini et al., 2017). The main signals in the aromatic region of the HSQC spectrum are attributed to the substituted aromatic rings of the kraft lignin units. The spectrum is predominated by the strong signals of guaiacyl (G) units at C2–H2 (δC/δH 109.5-112.8/6.7–7.2 ppm), C5–H5 (δC/δH 115.3/6.8 ppm), and C6–H6 (δC/δH 118.7–121.8/6.5–7.3 ppm). The kraft lignin of the present study was originated by a softwood (spruce), which is known to contain mainly coniferyl (guaiacyl, G units) alcohol units as lignin precursor (Azadi et al., 2013). A smaller signal related to cinnamaldehyde (Jβ, 125.6/7 ppm) was also observed, and maybe due to aldehydes produced during the kraft pulping, in low amounts (Crestini et al., 2017).

The main signals in the part of the spectrum with the inter-unit linkages correspond to the common structures of β-O-4′ aryl ethers, resinol substructures β-β′, and the phenylcoumaran substructures β-5′ linkages (Table S1). These linkages represent the major internal connections, which construct the three-dimensional structure of lignin. More specifically, the α-, β- and strong signal at γ-position of β-O-4′ (type A) linkages are shown at δC/δH 71/4.8, 84.1/4.3, and 60.0/3.4 ppm, respectively. In addition, the signals of α, β and γ C atoms of resinol structures (β-β′ linkages, type B) are observed in the spectrum at δC/δH 84.9/4.6, 53.5/3.1, and 70.9/4.16 ppm respectively. The phenylcoumaran (β-5′, type C) units are also identified on the spectrum, and the signals of α and γ carbon atoms are observed at δC/δH 86.8/5.5 and 60–60.3/3.8 ppm, respectively. Moreover, a small peak that correspond to cinnamyl alcohol end groups (I) are also observed by their Cγ-Hγ correlations at δC/δH 61.5/4.1 ppm. For the calculation of the ratio of the different types of inter-unit linkages, the cross-peaks that correspond to the α atoms of each type of linkage were integrated, except for the peaks of J and I structures for whom the peaks of β and γ C atoms were used, respectively. The resulting ratio of A, B, C, J, I structures was 25.4/2.4/1.2/6.4/1.2 respectively, based on the area of G2 units that was set as 100 Ar. Apart from the cross-peaks that correspond to α, β, γ C atoms of A, B, C types of interunit linkages, there is also the above mentioned cross-peak at 125.6/7.0 that some studies (Yuan et al., 2011; Wen et al., 2013; Crestini et al., 2017) assigned to the β atom of the cinnamaldehyde (J structure) and some other (for example Constant et al., 2016) to the α, β C atoms of the stilbene structure. These two structures are similar and both have a double bond in conjunction with the aromatic ring. The existence of this peak implies that such double bond exists in our kraft lignin sample in addition to the A, B, C types of inter-unit linkages. The existence of J structure is also evidenced from the presence of the cross-peak at 61.5/4.1 on the linkage part of the spectrum that is assigned to the γ atom of cinnamyl alcohol (I) which is the reduced form of the J structure and exists in a smaller extent.

### Physicochemical characteristics of the ZSM-5 catalysts

**Figure 3:**
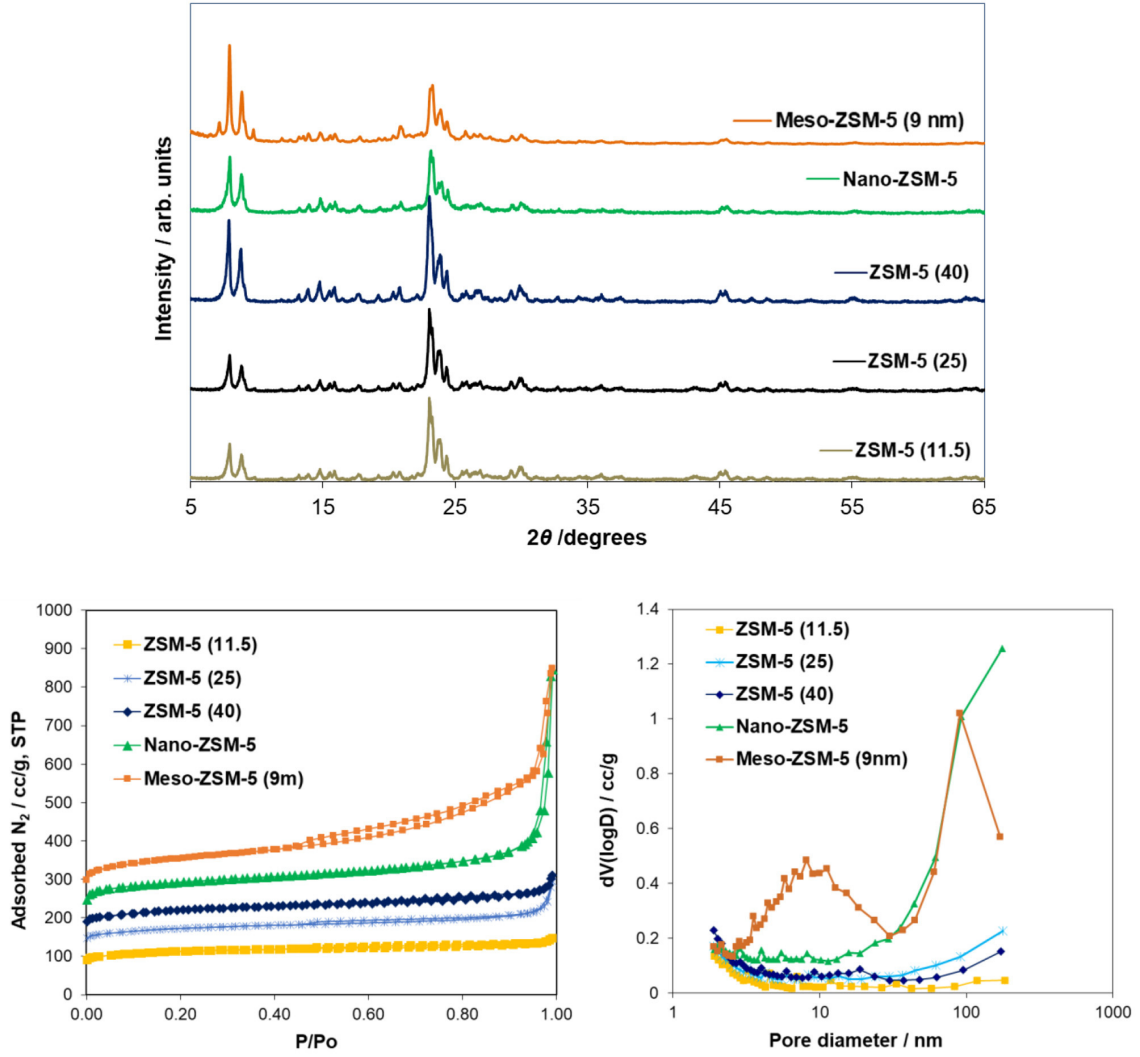
X-ray diffraction patterns (XRD) and N_2_ adsorption-desorption isotherms of the conventional microporous ZSM-5 (Si/Al = 11.5, 25, 40) zeolites, the nanosized ZSM-5 zeolite synthesized under controlled/mild template hydrothermal synthesis, and the mesoporous ZSM-5 (9nm) zeolite prepared by mild alkaline treatment of the conventional ZSM-5(40) zeolite.

The XRD patterns of the ZSM-5 zeolite catalysts used are shown in Figure 3. The patterns of all the samples (commercial microporous, meso- and nano-ZSM-5 zeolites) showed the characteristic diffraction peaks of the MFI crystalline structure. However, a slight decrease of the peak intensity in the range of ca. 20–25° 2θ was observed for the nano-sized and mesoporous ZSM-5 samples, as well as a very broad and low intensity bump in the pattern of the mesoporous ZSM-5 prepared by alkaline treatment, due probably to the presence of small amounts of amorphous silica phases.

**Figure 4:**
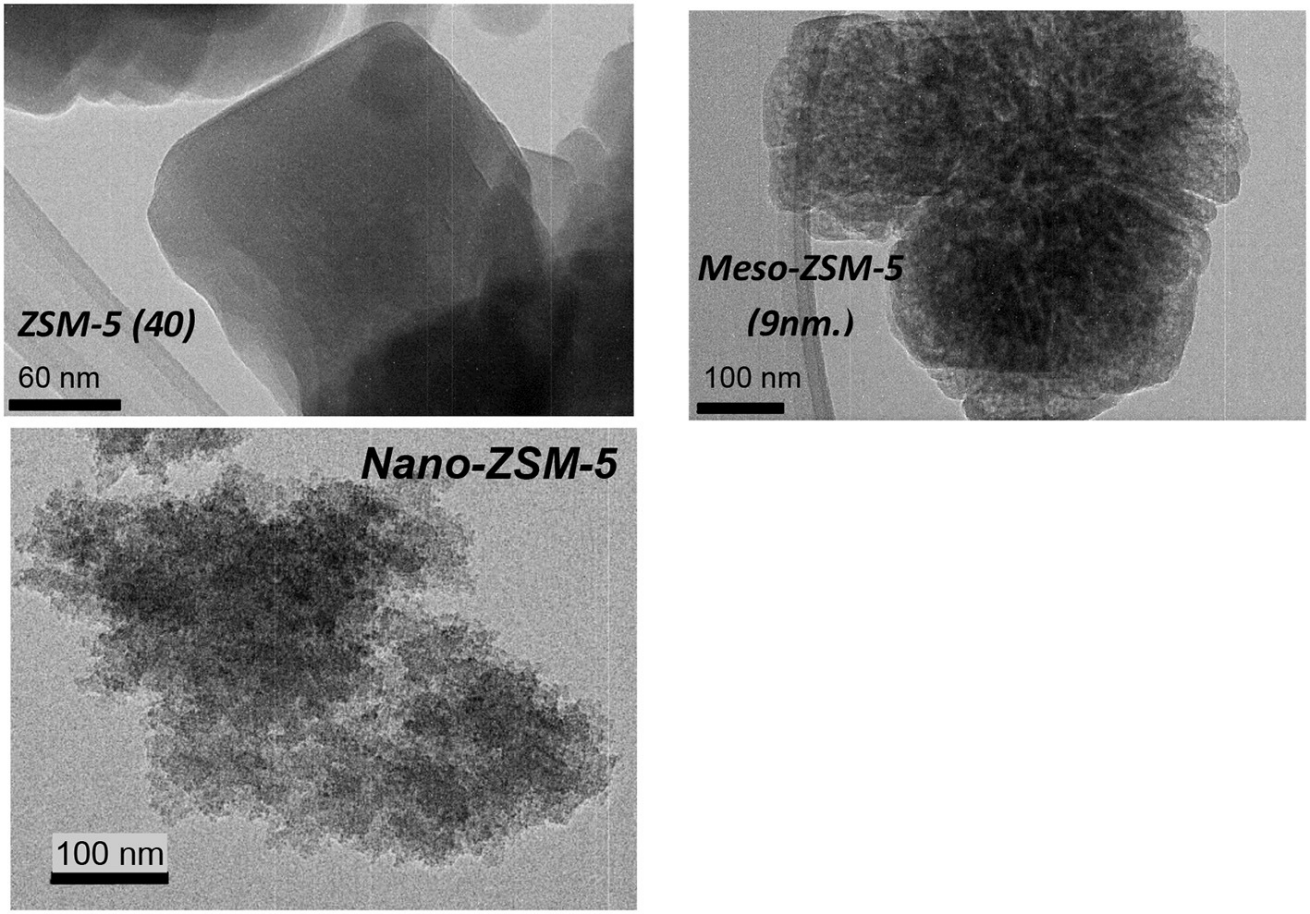
Transmission electron microscopy (TEM) images of the conventional microporous ZSM5 (40) zeolite, the nanosized ZSM-5 and the alkaline-treated mesoporous ZSM-5 zeolite.

The N_2_ adsorption-desorption isotherms and the BJH pore width distribution curves of the ZSM-5 zeolite catalysts are shown in Figure 3. The conventional microporous ZSM-5 zeolites with different Si/Al ratio exhibit the type I(a) adsorption isotherm according to the updated IUPAC classification (Thommes et al., 2015), with a plateau at higher relative pressures and no distinct hysteresis loop, typical for microporous zeolitic materials without significant mesoporosity. The BET area of the conventional microporous ZSM-5 zeolites ranged between 425 and 455 m^2^/g with about 330–350 m^2^/g being attributed to microporous area (t-plot method) (Table 2). The absence of any intra-crystal defects or voids which may induce increased meso/macroporosity was evidenced by the well-formed crystals observed in their TEM images. A representative image of ZSM-5(40) is shown in Figure 4 where relatively large “plate”-like crystals of parallelepiped shape typical for the MFI zeolites can be seen.

**Table 2:** Porosity, chemical composition and acidity data of the various ZSM-5 zeolite catalysts.

**Catalyst**	**BET area^a^ (m^2^/g)**	**Micro- pore area^b^ (m^2^/g)**	**Meso/ macro pore & external area^c^ (m^2^/g)**	**Average mesopore diameter^e^ (nm)**	**Chemical composition**^**f**^		**Acidity**^**g**^		
					**Al**	**Na**	**FT-IR/pyridine (**μ**mol Pyr/g)**		
					**(wt.%)**		**Brønsted**	**Lewis**	**B/L**
ZSM-5(11.5)	424	349	75	–	3.20	0.06	430	123	3.5
ZSM-5(25)	456	350	106	–	1.50	0.03	216	46	4.7
ZSM-5(40)	437	332	105	–	0.91	0.03	190	26	7.3
Meso-ZSM-5 (9nm)	560	259	301	9.0	0.82	0.05	192	21	9.1
Nano-ZSM-5	524	343	181^d^	–	0.86	0.08	100	53	1.9

a *BET area from N_2_ sorption at−196°C, Multi-point BET method*.

b *t-plot method*.

c *Difference of BET area minus micropore area*.

d *Attributed mainly to macropores and external surface area*.

e BJH analysis using adsorption data;

f *ICP-AES chemical analysis data*.

g *Determination of the amount of Brønsted and Lewis acid sites performed by Fourier transform – infrared (FT-IR) spectroscopy combined with in situ adsorption of pyridine*.

The N_2_ adsorption isotherm of the nano-sized ZSM-5 were also of type I(a) with an additional feature at high relative pressures (P/Po ≥ 0.9) where a steep increase of adsorbed nitrogen was observed, indicating high macroporous/external surface area due to the very small crystallites/particles of this zeolite. This was confirmed by the TEM image (Figure 4) of nano-ZSM-5 which revealed the presence of aggregated primary nanocrystals of less than 20 nm in size, generating inter-crystal (textural) porosity and high macroporous/external surface area. As a result, the nano-ZSM-5 zeolite possesses equally high micropore area with the conventional microporous zeolites and additional increased macropore/external surface area (Table 2). In the case of meso-ZSM-5 (9 nm) prepared by mild alkaline treatment followed by treatment with dilute acid aqueous solution, the N_2_ adsorption isotherm was of combined type I(a) and II, indicating the presence of both micropores and mesopores with very broad width distribution. Indeed, as can be seen in the BJH curves of Figure 3, the pore width distribution of meso-ZSM-5 exceeds from ca. 2.5 to 30 nm, with a maximum at about 9 nm. Such broad distribution of the size of intra-crystal mesopores is typical for meso-ZSM-5 zeolites prepared by mild alkaline treatment, compared to ZSM-5 zeolites synthesized by the use of a meso-structure directing agent (Choi et al., 2006; Groen et al., 2006; Park et al., 2009; Gamliel et al., 2016). The significant increase of the meso/macroporous and external area is also accompanied by a substantial decrease of the microporous area (Table 1). This effect is often observed in mesoporous ZSM-5 zeolites with intra-crystal mesoporosity (Park et al., 2009; Gamliel et al., 2016) and can be attributed to partial disordering of the mesopore walls, not contributing to zeolitic microporosity, as well as to the presence of small amounts of amorphous silica-alumina impurities. The partial dissolution of the zeolitic silicate framework, the consequent removal of Al atoms and the “healing” of the Si-OH nests lead to the formation of “secondary” cavities and pores in the crystal, as can be seen in the representative TEM image of meso-ZSM-5(9 nm) in Figure 4.

Representative Ar physisorption data are shown in Figure S3 and Table S2 in Supplementary Material. Small differences (< 10%) in the BET (total) and micropore areas derived from the two probes (N_2_ and Ar) were observed. On the other hand, a noticeable difference was found in the determination of the average mesopore width of the mesoporous ZSM-5 zeolite when applying different analysis methods (~9nm from N_2_ and Ar—BJH, ~6.5 from N_2_ NLDFT and ~5.7 nm from Ar NLDFT). Still, the formation of intra-crystal mesoporosity, with relative broad width distribution, in the meso-ZSM-5 zeolite prepared by mild alkaline treated was clearly shown.

The acidic properties of zeolite catalysts are of high importance when they are intended for use in biomass or petroleum cracking/pyrolysis. ZSM-5 zeolite is considered as a relatively strongly acidic zeolite containing mainly Brønsted acid sites, if no extra-framework amorphous phases are present (Triantafillidis et al., 2001; Triantafyllidis et al., 2004; Komvokis et al., 2012). As shown in Table 2, the number of Brønsted acid sites decreases with increasing Si/Al of the commercial microporous ZSM-5 zeolites; however, the content of Lewis acid sites follows the reverse trend and increases with increasing Al content, i.e., lower Si/Al ratio. This is attributed to the easier framework dealumination of Al-rich zeolites during the calcination procedures that are subjected to, i.e., for removing the organic template after synthesis and for converting the ${\text{NH}}_{4}^{+}$-form to H^+^-form. Overall, the ratio of Brønsted to Lewis acid sites increases with Si/Al ratio. Furthermore, as can be seen in Figure S4 (Supplementary Material), the Brønsted acid sites of ZSM-5(11.5) are of lower relative strength compared to those of ZSM-5(25) and ZSM-5(40). In the case of meso-ZSM-5 (9 nm) prepared by mild alkaline treatment followed by treatment with the dilute HCl aqueous solution, the content of Brønsted sites and the B/L ratio remain high, thus showing that the acidic properties of ZSM-5 are not sacrificed by this methodology. The relatively low content of Lewis acid sites is attributed to the “cleaning” of the alkaline-treated sample from the extra-framework aluminum phases by the use of the dilute HCl solution. The nano-ZSM-5 zeolite, despite having similar Al content with the ZSM-5(40) zeolite (Table 2), contained almost half of its Brønsted sites, possibly due to the relatively lower degree of crystallinity and inadequate organization of the zeolitic framework. The relatively higher content of Lewis acid sites may further confirm this hypothesis.

### Non-catalytic and catalytic fast pyrolysis of kraft lignin (Py/GC-MS)

#### Thermal pyrolysis of kraft lignin at various temperatures (Py/GC-MS system)

**Figure 5:**
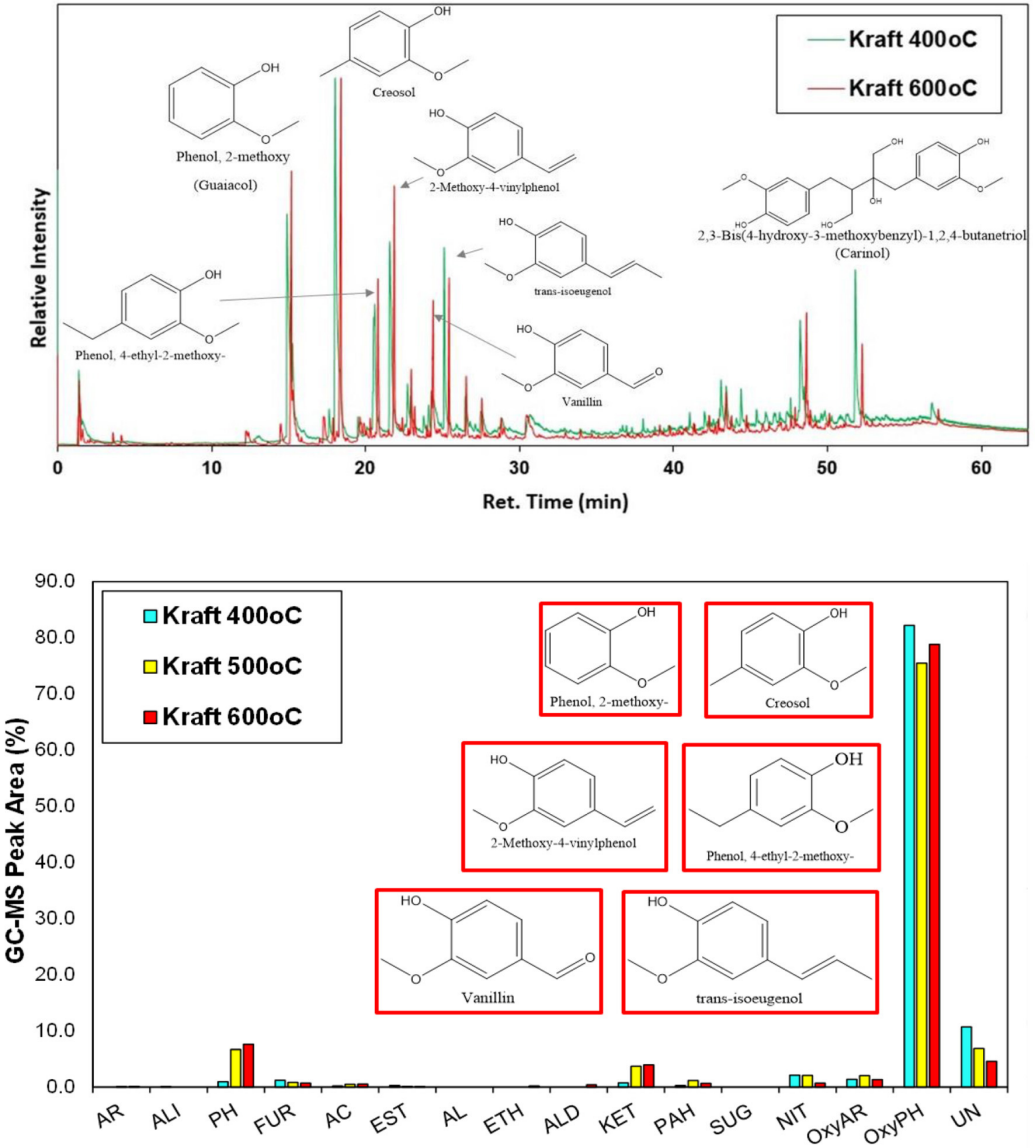
Representative Py/GC-MS spectra of non-catalytic (thermal) fast pyrolysis of kraft (spruce) lignin (up), and relative concentration of the various groups of compounds in the bio-oil (down); indicated compounds in the graph are for the experiment at 600°C.

Non-catalytic fast pyrolysis tests using silica sand as inert heat carrier were conducted on the Py/GC-MS system at 400, 500, and 600°C. Representative chromatograms at 400 and 600°C are presented in Figure 5, while detailed list of the identified compounds at all three temperatures is given in Table S3 (Supplementary Material). The lignin pyrolysis vapors comprised mainly of alkoxy-phenols with single alkoxy group, i.e., of the guaiacol (G) type, such as guaiacol (2-methoxy phenol), creosol, 2-methoxy-4-vinylphenol, 4-ethyl-2-methoxy phenol, vanillin and trans-isoeugenol. This clearly indicates that the predominant G units identified by 2D HSQC NMR in the structure of lignin were “transferred” to the produced bio-oil vapors. At 400°C, trace amounts of oxy-aromatics, ketones, furans and alkyl-phenols (not oxygenated) were also produced in addition to the alkoxy-phenols.

It is has been previously shown that the increase of the pyrolysis temperature promotes the decomposition of high molecular weight phenolic oligomers to smaller compounds via depolymerisation and cracking of C-C bonds (Jiang et al., 2010; Wang et al., 2014). In the present work, this effect was only slightly identified as by increasing the pyrolysis temperature from 400 to 500, and 600°C the concentration of alkoxy-phenols decreased by ca. 5% giving rise to more alkyl-phenols (increase by 6–7%). Unidentified compounds, usually being high molecular weight fragments, were also decreased. Thermal pyrolysis of lignin is also known to produce relatively high amounts of solid carbonaceous material (char), due to recondensation/repolymerization of primary pyrolysis oligomers as well as condensation and coupling reactions of guaiacol and other small alkoxy-phenols (Gayubo et al., 2004; Wang et al., 2009, 2014; Stefanidis et al., 2014; Custodis et al., 2016). The char produced by pyrolysis of kraft lignin at 400°C was 54.5 wt.% and decreased to 43.3 wt.% at 500°C and 34.5 wt.% at 600°C (Table S3), thus indicating that higher temperatures, at least in the range of 400–600°C, favor the formation of low molecular weight species instead of polymerized products and char.

#### Catalytic fast pyrolysis of lignin with ZSM-5 zeolite: effect of temperature and C/L ratio (Py/GC-MS system)

**Figure 6:**
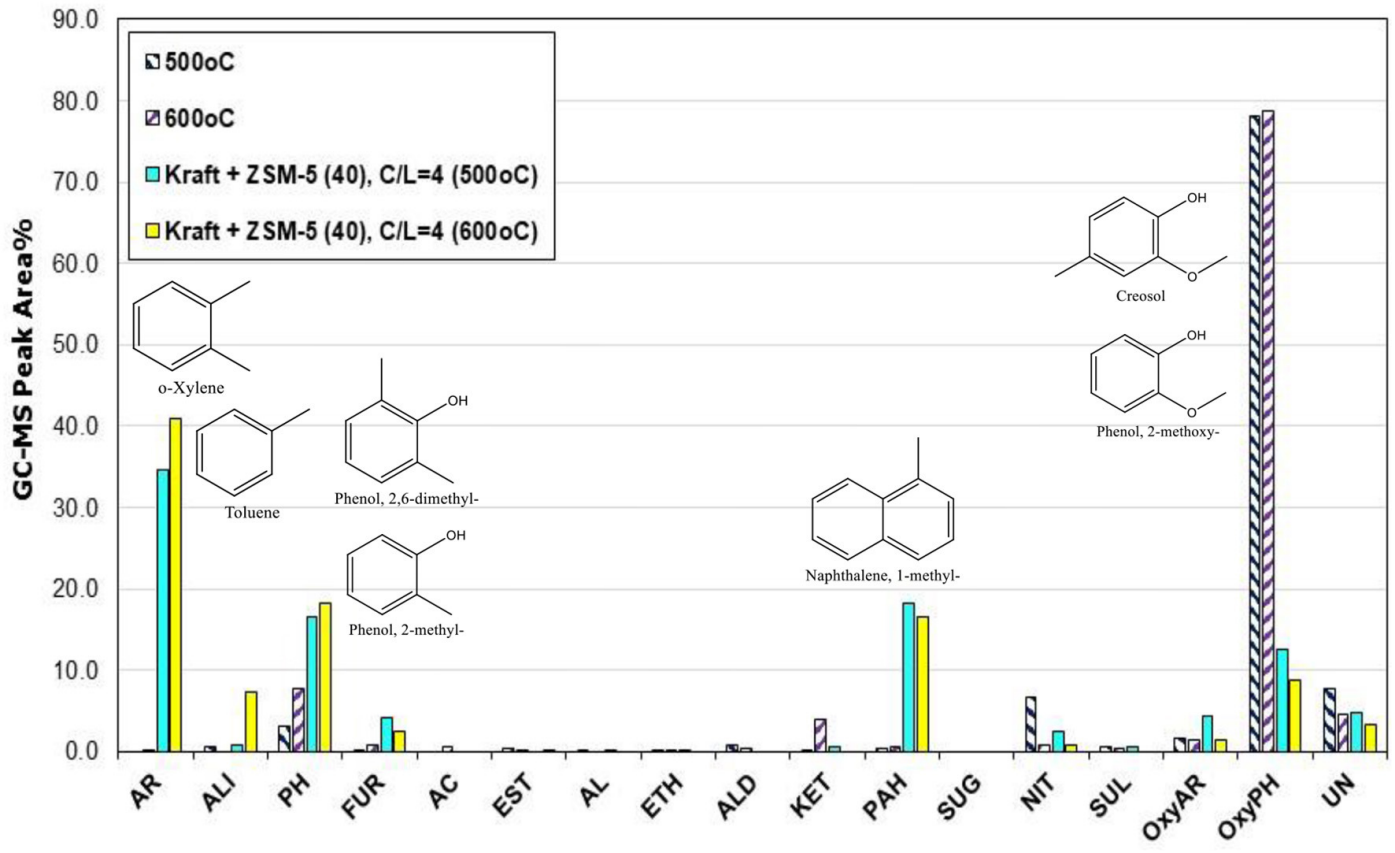
Relative concentration of the various groups of compounds in the bio-oils derived by catalytic fast pyrolysis (CFP) of kraft lignin with ZSM-5(40) zeolite at two temperatures (500 and 600°C) and catalyst-to-lignin (C/L) ratio of 4; indicated compounds in the graph refer to the experiment at 600°C.

**Figure 7:**
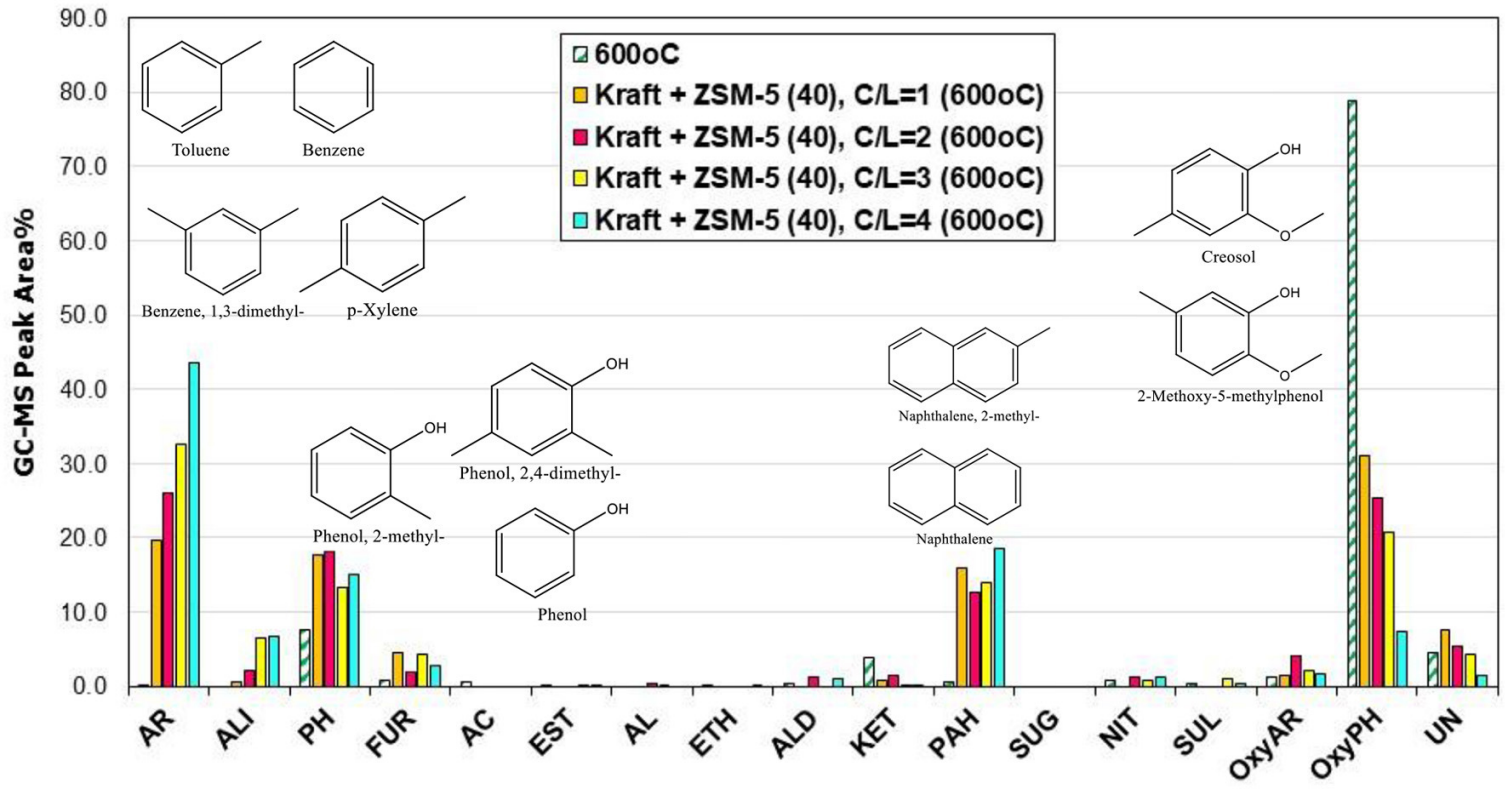
Relative concentration of the various groups of compounds in the bio-oils derived by catalytic fast pyrolysis (CFP) of kraft lignin with ZSM-5(40) zeolite at 600°C and at different catalyst-to-lignin (C/L) ratios of 1, 2, 3, and 4; indicated compounds in the graph refer to the experiment at C/B = 4.

The catalytic fast pyrolysis (CFP) tests in the Py/GC-MS system were performed with the different ZSM-5 zeolites described in the previous section. The effect of pyrolysis temperature and catalyst-to-lignin (C/L) ratio was initially investigated by the use of the conventional microporous zeolite ZSM-5(40). As a general observation, the use of the ZSM-5 zeolite induced the substantial conversion of alkoxy-phenols toward mono-aromatics (BTX, such as benzene, toluene, p-xylene, o-xylene, 1,3-dimethyl-benzene, etc.), PAHs (mainy naphthalenes) and alkyl-phenols, such as phenol, 2-methyl-phenol, 2,3-dimethyl-phenol, etc. (Figure 6 and Table S2), in accordance with previous related works on the use of zeolites and especially ZSM-5 in lignin pyrolysis (Jackson et al., 2009; Li et al., 2012; Ben and Ragauskas, 2013; Custodis et al., 2016). By increasing the pyrolysis temperature from 500 to 600°C, this effect was more pronounced favoring mostly the mono-aromatics and the naphthalenes (Figure 6). The higher C/L ratio had also a beneficial effect on the conversion of alkoxy-phenols toward mono-aromatics and PAHs, both at 500°C (Figure 6) as well as at 600°C (Figure 7). At the more intense conditions, i.e., C/L = 4 and temperature of 600°C, a highly deoxygenated bio-oil can be produced which contains mainly aromatics as well as some alkyl-phenols. The concentration of alkyl-phenols appears to be less depended on the pyrolysis temperature and the C/L ratio, exhibiting a nearly constant value for all C/L and temperatures tested.

In addition to the char formed in thermal (non-catalytic) pyrolysis of lignin, in the case of CFP coke is also formed via condensation/polymerization reactions on the acid sites of the zeolites. It has been suggested that thermally resistant lignin oligomers and the low reactivity of phenolics over zeolitic catalysts may be the reasons for enhanced formation of char/coke, in addition to the more classical polymerization of aromatics and PAHs (Wang et al., 2014). The char/coke obtained with zeolite ZSM-5(40) as catalyst, at 500°C and C/L = 2 ratio, was 44.6 wt.% and was slightly higher compared to thermal (non-catalytic) char at 500°C (43.3 wt.%), as can be seen in Table S4 (Supplementary Material). The same trend was observed at 600°C, at C/L = 2, when the thermal char and catalytic char/coke were compared. However, at both temperatures, when the amount of zeolite catalyst was increased (C/L = 4), the amount of char/coke was markedly lower in the catalytic tests compared to the non-catalytic thermal pyrolysis, i.e., from 43.3 and 34.5% to 39.9% and 28.1% at 500 and 600°C, respectively (Table S4). This can be attributed to the increased conversion of intermediate lignin oligomers toward aromatics and alkyl-phenols on the active acid sites of ZSM-5 and the balanced/suppressed formation of reaction coke via polymerization. Similar results were reported previously by the use of ZSM-5 and USY catalysts in alkali lignin pyrolysis (Ma et al., 2012; Custodis et al., 2016).

#### Catalytic fast pyrolysis of lignin with ZSM-5 zeolite: effect of Si/Al (Py/GC-MS system)

**Figure 8:**
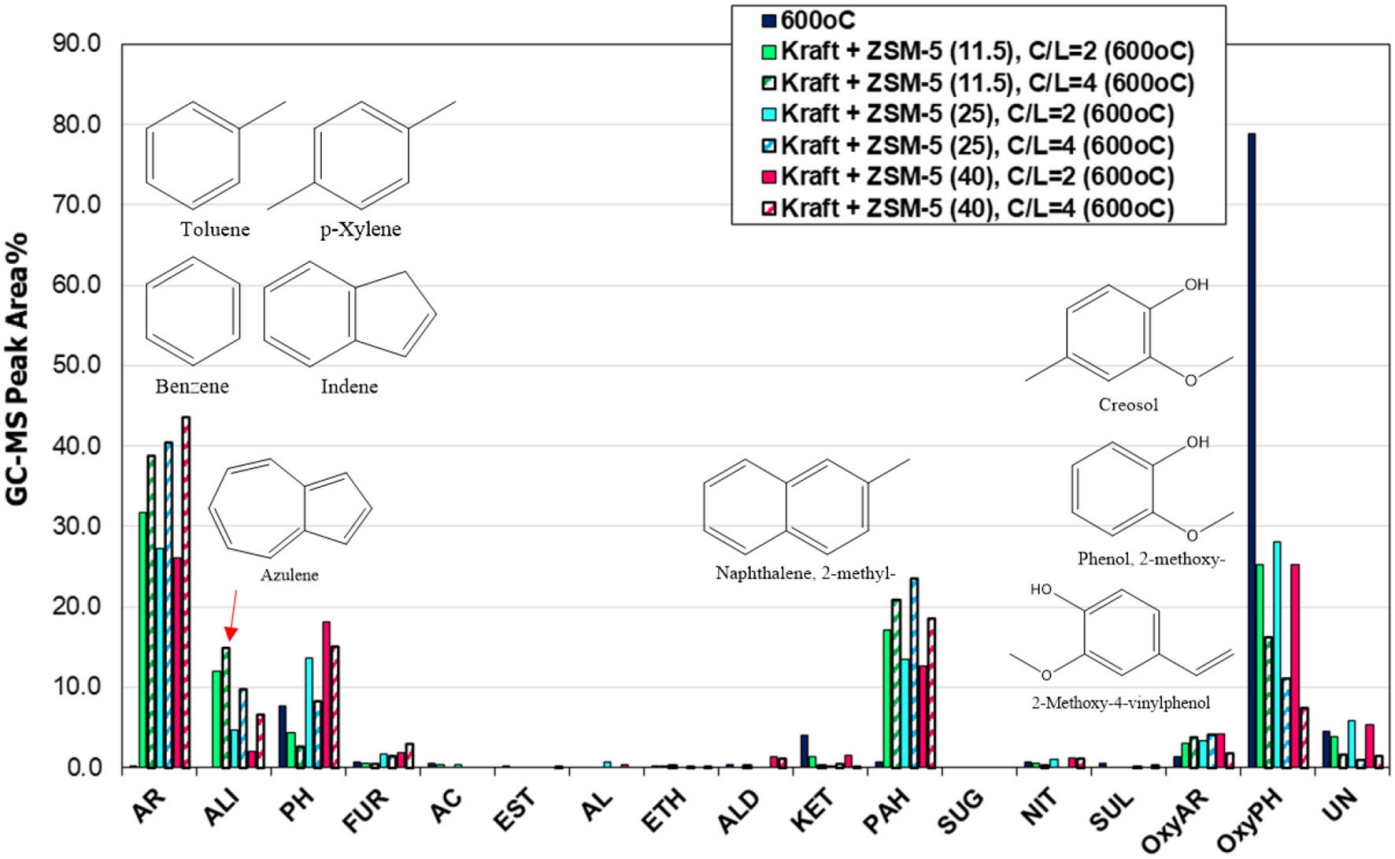
Relative concentration of the various groups of compounds in the bio-oils derived by catalytic fast pyrolysis (CFP) of kraft lignin with ZSM-5 zeolites having different Si/Al ratio (11.5, 25, and 40) at 600°C and at different catalyst-to-lignin (C/L) ratios (2 and 4); indicated compounds in the graph are for ZSM-5 (11.5) at C/L = 4.

**Figure 9:**
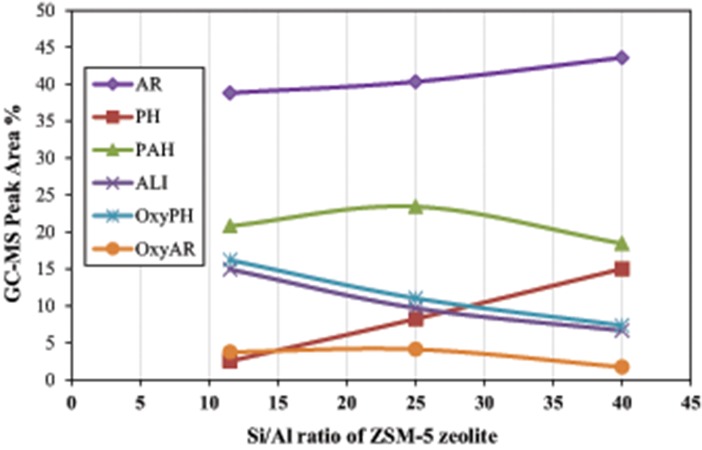
Correlation between the Si/Al ratio of ZSM-5 zeolite with the relative concentration of the most representative groups of compounds in the bio-oil derived by catalytic fast pyrolysis (CFP) of kraft lignin at 600°C and C/L = 4 (AR, mono-aromatics; PH, phenol and alkyl-phenols; PAHs, polycyclic aromatic hydrocarbons; ALI, aliphatic hydrocarbons, OxyPH, oxygenated phenolics; OxyAR, oxygenated aromatics).

The acidity of the zeolitic catalysts, mainly the Brønsted acid sites, are responsible for the deoxygenation of the bio-oil via dehydration, decarbonylation and decarboxylation reactions, as well as for the enhanced formation of aromatics either via deoxygenation of the phenolics or via aromatization of the small alkenes C2 = and C3 = formed initially via thermal or catalytic cracking (Mullen and Boateng, 2010; Ma et al., 2012; Ben and Ragauskas, 2013; Wang et al., 2014). The amount and strength of acid sites, in combination with the specific micropore framework structure of each zeolite type, have a pronounced effect on the reactions that occur during the pyrolysis process and the final product yields and bio-oil composition. For this reason, we investigated the effect of Si/Al ratio (11.5, 25, and 40) of ZSM-5 zeolite on the composition of bio-oil via the Py/GC-MS lignin pyrolysis tests. Many studies suggested that when the Si/Al ratios of ZSM-5 decreased and the amount of acid sites was increased (due to higher aluminum content), the yields of phenolics and other oxygenates progressively declined, while the yields of aromatics increased substantially (Li et al., 2012; Ma et al., 2012; Yu et al., 2012). In the present work, we examined the effect of Si/Al ratio of ZSM-5 at 600°C and at two C/L ratios, i.e., 2 and 4. The relative concentration of the various groups of compounds produced is shown in Figure 8. At C/L = 2 all three ZSM-5 zeolites induced a similar conversion/decrease of alkoxy-phenols, while the ZSM-5(11.5) sample with the higher content of Brønsted acid sites (Table 2) exhibited an enhanced formation of aromatics and some aliphatics (mainly azulene) compared to the other two ZSM-5's, in accordance with the previous works. However, when using higher amount of catalyst (C/L = 4), the ZSM-5(25) and especially the ZSM-5(40) zeolite were more reactive in converting the alkoxy-phenols toward mono-aromatics as well as alkyl-phenols compared to ZSM-5(11.5). This indicates the beneficial effect of using higher amount of Brønsted acid sites of higher strength, since ZSM-5(40) possessed fewer but relatively stronger acid sites compared to ZSM-5(11.5), as can be revealed by the FT-IR/sorbed pyridine measurements at increasing pyridine equilibration temperature, i.e., 150–450°C (data shown in Figure S4, Supplementary Material). Thus, it can be suggested that the bio-oil can be enriched in mono-aromatics and alkyl-phenols when higher amount of ZSM-5 zeolite (higher C/L ratio) with relatively high Si/Al ratio (ca. 40) and fewer but stronger acid sites is used. The effect of Si/Al ratio of ZSM-5 on the most important groups of compounds in bio-oil is more clearly presented in Figure 9.

#### Catalytic fast pyrolysis of lignin with conventional microporous, nano-sized and mesoporous ZSM-5 zeolites (Py/GC-MS system)

**Figure 10:**
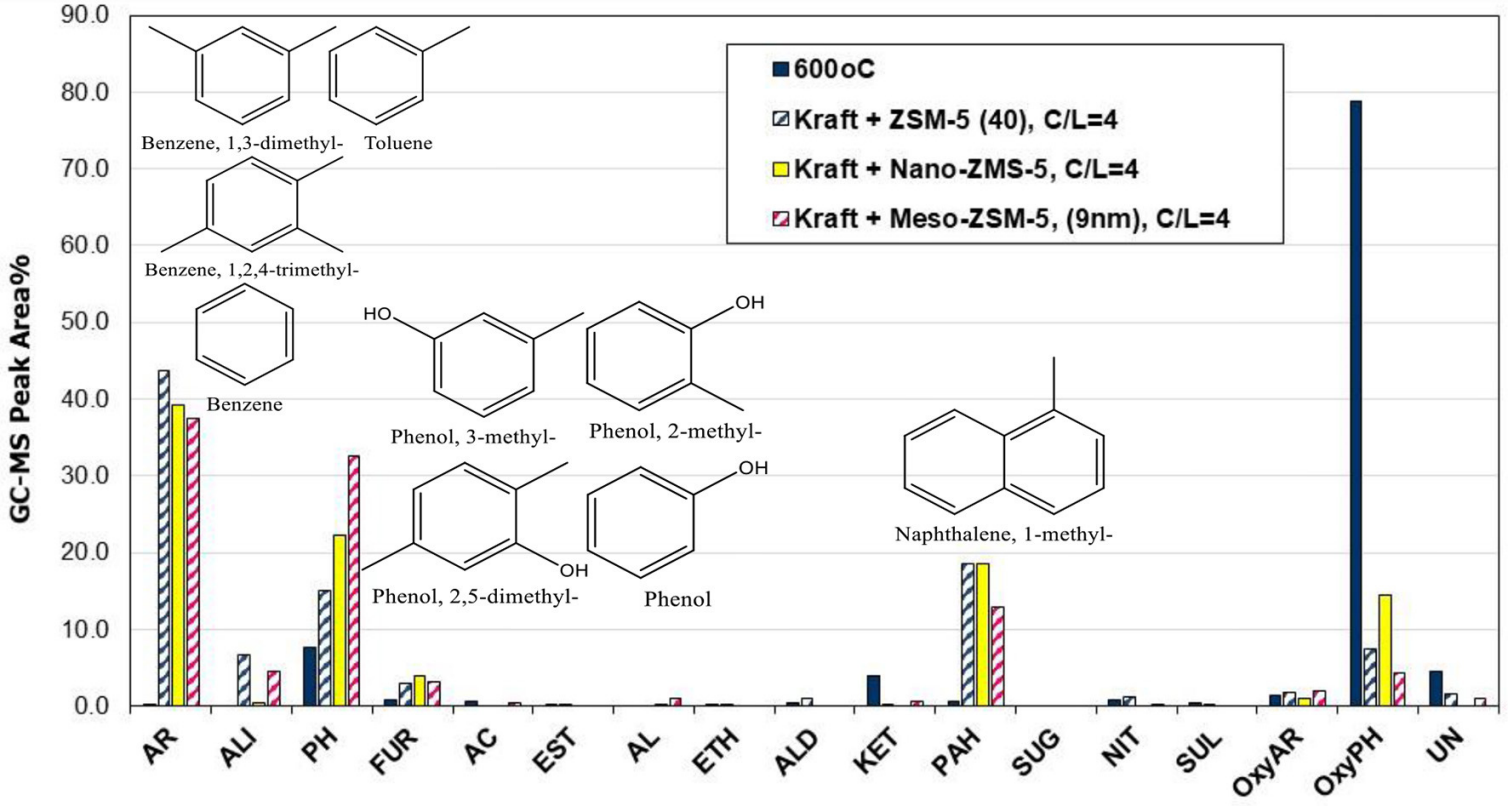
Relative concentration of the various groups of compounds in the bio-oils derived by catalytic fast pyrolysis (CFP) of kraft lignin with conventional microporous, nano-sized and mesoporous ZSM-5 zeolites at 600°C and at catalyst-to-lignin C/L = 4; indicated compounds in the graph are for the mesoporous ZSM-5 (9 nm).

The effect of porous characteristics and crystal/particle size of ZSM-5 zeolite on the composition of bio-oil was investigated by using the most reactive (based on the above comparisons) conventional microporous ZSM-5(40) zeolite, a synthesized nano-sized ZSM-5, and a mesoporous ZSM-5 prepared via alkaline treatment of the ZSM-5(40) zeolite. The aluminum content (and Si/Al ratio) of all three zeolites was similar (Table 2); however, they differed in the amount of acid sites as the nano-sized zeolite contained almost half Brønsted acid sites compared to the other two zeolites. Considering also its relatively higher amount of Lewis acid sites, this can be attributed to incomplete crystallization of this zeolite, despite the clear and strong reflections shown in its XRD pattern (Figure 3). The Py/GC-MS lignin pyrolysis results (at 600°C and C/L = 4) with the three zeolites are shown in Figure 10. All catalysts were very reactive in converting the alkoxy-phenols toward mono-aromatics, naphthalenes (PAHs) and alkyl-phenols. Interestingly, the nano-sized ZSM-5, despite its significantly lower amount of Brønsted acid sites, exhibited similar reactivity/performance compared to the other two zeolites, thus proving the beneficial effect of the higher accessibility of this zeolite due to its small crystal/particle size and the high textural/interparticle porosity (Table 2, Figures 3, 4). However, the char/coke production by the nano-sized zeolite was higher (about 33.4 wt.%) compared to that by the microporous ZSM-5(40) zeolite (28.1 wt.%) (data shown in Table S4), thus indicating that condensation and polymerization reactions of mono-aromatics or phenolics are facilitated on the acid sites located in the intra-particle voids (high textural porosity of nano-sized ZSM-5). Similar trends of increased char/coke production by the use of nano-sized ZSM-5 with ca. < 100 nm crystal size, have been observed in catalytic pyrolysis of lignocellulosic biomass (Zheng et al., 2014; Gamliel et al., 2016).

The mesoporous ZSM-5 (9 nm) zeolite prepared by mild alkaline treatment, possessed similar acidic properties with the parent ZSM-5(40) zeolite (Table 2) and its performance in the pyrolysis of lignin was very promising. More specifically, as can be seen in Figure 10, it exhibited the highest reactivity in converting the alkoxy-phenols, the highest concentration of alkyl-phenols (phenol, 2-methyl-phenol, 3-methyl-phenol, 2,5-dimethyl-phenol, etc.), slightly less mono-aromatics (benzene, toluene, 1,3-dimethyl-benzene, 1,2,4-trimethyl-benzene, etc.), as well as fewer PAHs (1-methyl-naphthalene, 2,6-dimethyl-naphthalene, etc.). A possible reason for the lower formation of aromatics could be the reduction of its microporosity (with parallel increase of the mesoporosity) compared to the parent ZSM-5(40) zeolite, thus not promoting the aromatization reactions that take place on the Brønsted sites within the tubular micropores of ZSM-5. Interestingly, the char/coke produced by the mesoporous ZSM-5 (9 nm) zeolite (30.3%) was less compared to that with the nano-sized ZSM-5 (33.4%) and closer to that with the parent microporous ZSM-5(40) (28.1%) (Table S3). Previous works on biomass or lignin fast pyrolysis have reported either increased (Park et al., 2010; Foster et al., 2012) or decreased (Kelkar et al., 2014; Li et al., 2014; Gamliel et al., 2016) char/coke formation by the use of mesoporous ZSM-5 zeolites compared to conventional microporous ZSM-5. However, a general trend is difficult to be established as coking depends on pyrolysis temperature, mesopore width and acidic properties, catalyst to biomass/lignin ratio, type of feedstock and type of experimental set-up (micro-pyrolyzer vs. bench scale fixed bed units, etc.), as discussed also below.

### Non-catalytic and catalytic fast pyrolysis of kraft lignin (fixed-bed unit)

**Figure 11:**
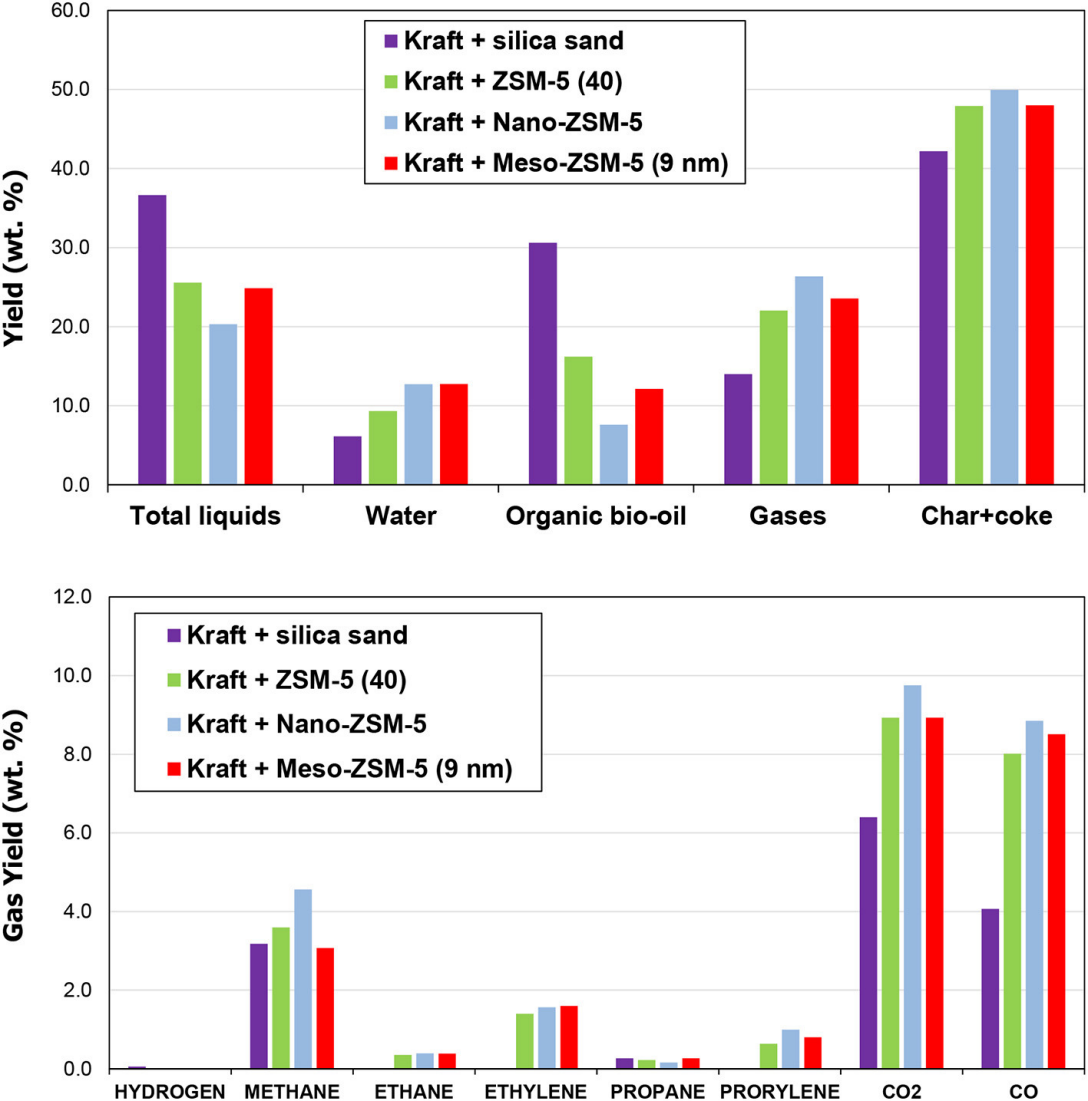
Product yield distribution and gas analysis from the thermal and catalytic fast pyrolysis of kraft lignin (results from the fixed bed unit).

The three ZSM-5 zeolites, i.e., the conventional microporous ZSM-5(40), the nano-sized ZSM-5 and the mesoporous ZSM-5(9nm), were also evaluated in the fixed bed unit described in the experimental section and in Supplementary Material. The total liquids (bio-oil) yield in the non-catalytic test was 36.6 wt.% (6.1 wt.% being water and the remaining 30.6 wt.% comprising of organics), the non-condensable gases 14 wt.% and the solids (char) 42.4 wt.% (Figure 11 and Table S5 in Supplementary Material). By the use of conventional microporous ZSM-5(40) zeolite, the total liquids yield decreased to 25.6 wt.%, due to significant decrease of the organic fraction (16.2 from 30.6 wt.% in the thermal bio-oil). The non-condensable gases and the solids (char plus coke on catalyst in this case) increased to 22.0 and 47.9 wt.%, respectively. The corresponding changes in yield values were slightly enhanced by the use of mesoporous ZSM-5(9 nm), while in the case of nano-sized ZSM-5, the total liquids were further reduced to 20.3 wt.% (the organic fraction dropped to 7.6 wt.%) and the gases and solids were even higher (26.4 and 49.9 wt.%, respectively). Similar changes in the product yields have been previously reported by the use of conventional microporous or mesoporous ZSM-5 zeolites in biomass or lignin pyrolysis using fixed bed units where the initially formed biomass pyrolysis vapors reacted subsequently over the catalyst bed in the same reactor (Park et al., 2010; Stephanidis et al., 2011; Kalogiannis et al., 2015). On the other hand, in several studies based on micro-pyrolyzer systems either with tube (Ma et al., 2012; Custodis et al., 2016) or bucket (Mihalcik et al., 2011; Wang et al., 2014) form, the presence of the zeolite resulted in decrease of the solids (char plus coke) and in some cases increase of the bio-oil yield compared to the non-catalytic pyrolysis. In the present work, the Py/GC-MS experiments discussed in the previous section showed also a decrease of the solids formation upon use of either of the three ZSM-5 zeolites compared to the non-catalytic thermal pyrolysis (Table S4), in contrast to the results obtained from the fixed bed unit (Figure 11 and Table S5). However, the observed trend with regard to the effect of the type of ZSM-5 zeolite was the same for both experimental set-ups, i.e., the microporous ZSM-5(40) produced the lowest char plus coke and the nano-sized ZSM-5 the highest.

The increase of solids in the catalytic pyrolysis experiments is solely attributed to the coke formed on the catalysts, due to the design of the fixed bed reactor as described in the experimental section and in Supplementary Material. The data in Table S5 show that the formation of coke on the nano-ZSM-5 zeolite (7.7 wt.% on lignin) is clearly more pronounced compared to the conventional microporous ZSM-5 and the meso-ZSM-5(9 nm) (5.7 and 5.8 wt.% on lignin, respectively). The char derived from the thermal pyrolysis of lignin (as collected from the upper zone of the fixed bed reactor, Figure S2) and the coked catalysts (lower bed of the reactor, Figure S2), were further studied by thermogravimetric analysis (TGA); representative data are depicted in Figure S5 (Supplementary Material). It can be clearly seen that the thermal char decomposes under oxidative (air) atmosphere at lower temperature (DTG peak maximum at ~465°C) compared to the reaction coke formed on the zeolitic catalysts (DTG peak maximum at ~560–570°C), indicating different structure/composition of these two, and possibly the more condensed/carbonaceous nature of the coke on catalyst. However, more detailed characterization of the char and coke on catalyst are necessary in order to better understand their nature and associated mechanisms of formation.

The analysis of non-condensable gases is presented in Figure 11. The use of all three ZSM-5 zeolite catalysts induced the production of ethane, ethylene, propane and propylene which were not produced by the non-catalytic pyrolysis. The production of these gases is indicative of enhanced cracking and dealkylation reactions catalyzed by ZSM-5 in accordance with its performance in biomass fast pyrolysis (Mullen and Boateng, 2010; Stephanidis et al., 2011; Wang et al., 2014; Margeriat et al., 2018). As discussed below, ethylene and propylene serve as precursors for the production of aromatics in the channels of ZSM-5. The more abundant gases both in non-catalytic and catalytic lignin pyrolysis are methane, CO and CO_2_. With the use of the ZSM-5 zeolite catalysts both CO and CO_2_ were significantly increased due to decarbonylation and decarboxylation reactions, with CO being affected/increased to a higher extent. This is also typically observed in biomass CFP where ZSM-5 favors the formation of CO instead of CO_2_, as well as the formation of water via dehydration reactions (Stephanidis et al., 2011; Stefanidis et al., 2016).

**Figure 12:**
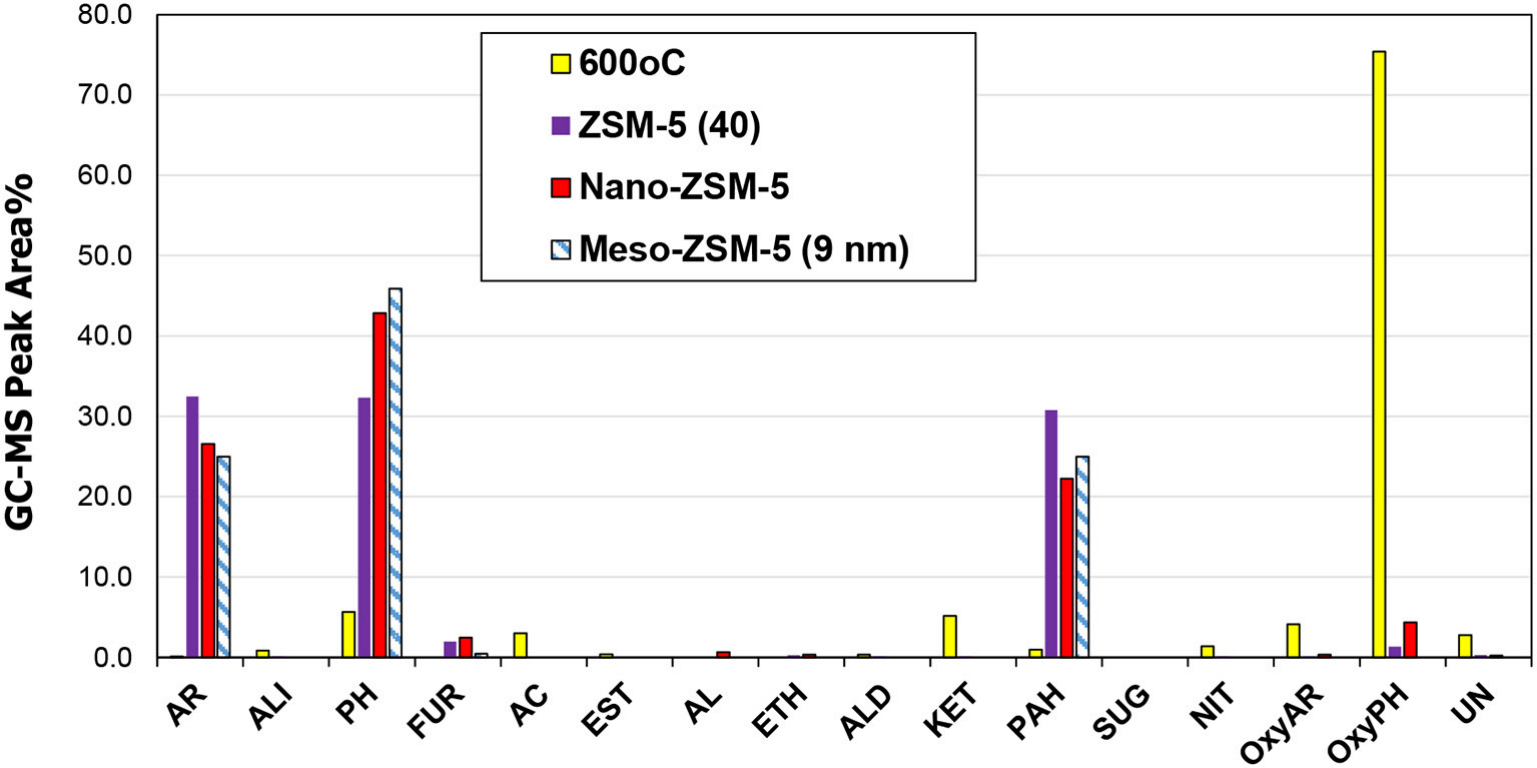
Relative concentration of the various groups of compounds in the bio-oils derived by non catalytic and catalytic fast pyrolysis (CFP) of kraft lignin with conventional microporous, nano-sized and mesoporous ZSM-5 zeolites (fixed bed unit, 600°C, C/L = 1).

The effect of the zeolite catalysts on bio-oil composition (Figure 12) was similar to that observed in the Py/GC-MS experiments (see results in previous section), although in the case of the fixed bed reactor the relative abundance between mono-aromatics and alkyl-phenols was in favor of the latter. More specifically, the conventional microporous ZMS-5(40) zeolite exhibited the highest selectivity toward aromatics formation, while the nano-sized ZSM-5 and especially the mesoporous ZSM-5(9nm) was more selective toward alkyl-phenols. These results are in line with the deoxygenation degree of the organic phase of bio-oil, as determined by elemental analysis, which was more pronounced by the use of the conventional ZSM-5 compared to the meso- and nano-ZSM-5 (Table S5, Supplementary Material). Nonetheless, all three ZSM-5 variants were quite effective in deoxygenating the bio-oil, inducing 50–60% reduction of oxygen compared to the oxygen of the thermal (non-catalytic) pyrolysis oil. However, it should be noted that the GC-MS analysis is capable of identifying only low molecular weight compounds and not bulkier oligomers usually present in thermal, non-catalytic bio-oils of biomass or lignin pyrolysis. Applying more advanced analytical techniques, such as 2DGC-ToFMS analysis, quantitative analysis of about 45% of the organic phase of lignin pyrolysis oil could be achieved, reaching as high as ~100% for the bio-oil obtained by the use of ZSM-5 zeolite catalyst (Kalogiannis et al., 2015).

Both the thermal and catalytic bio-oils contained also small amounts of sulfur, i.e., < 0.1 wt.% on lignin, thus indicating that most of the S of the parent kraft lignin (1.5 wt.%) has been “trapped” in the solids. Indeed, the elemental analysis of the thermal char and the coked catalysts showed the presence of about 0.3–0.6 wt.% S (Tables S5 and S6, Supplementary Material). Furthermore, sulfur in the gases (i.e., as H_2_S) was only scarcely identified at very low concentrations.

### Reaction mechanism and effect of ZSM-5 characteristics

**Figure 13:**
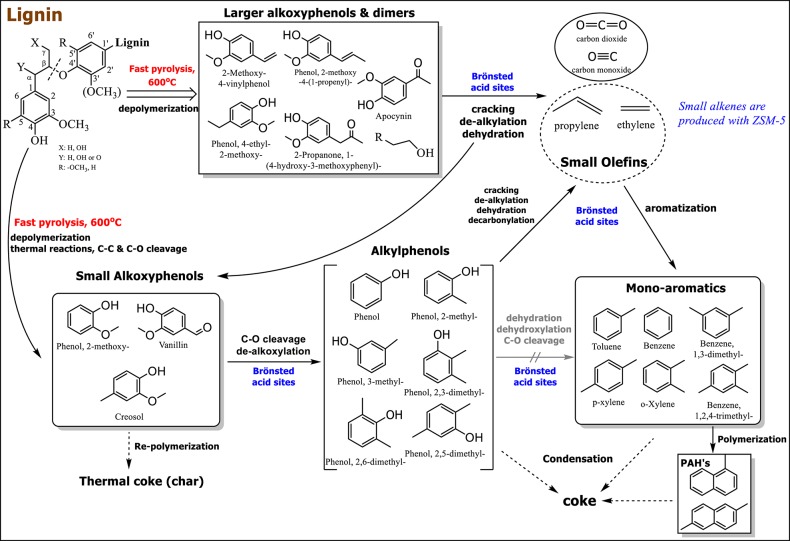
Possible pathways in non-catalytic and catalytic fast pyrolysis of kraft (softwood) lignin by the use of conventional microporous, mesoporous and nano-sized ZSM-5 zeolite.

Based on the analysis of the 2D HSQC NMR spectra (discussed above), as well as previous works on lignin characterization, the β-O-4 ether bond is the main linkage between lignin units isolated from softwood lignocellulosic biomass (i.e., spruce) (Sette et al., 2011; Chu et al., 2013; Du et al., 2013; Liu et al., 2015; Constant et al., 2016; Rinaldi et al., 2016). The relatively intense pyrolysis conditions, i.e., 600°C, provide the necessary energy for the thermal cleavage of the β-O-4 and α-O-4 ether bonds, thus providing the smaller oligomers and monomers that may react further on the acid sites of the zeolites (Figure 13). The non-catalytic Py/GC-MS results of the present work at different temperatures (400–600°C) revealed the formation of alkoxy-phenols with additional C2-C3 alkyl side chains as well as larger oligomers such as carinol (Figure 5 and Table S2). Thermal pyrolysis at higher temperatures, i.e., 600°C favored the formation of smaller alkoxy-phenols such as guaiacol, vanillin and creosol, as indicated in the mechanism of Figure 13. As suggested above and in accordance with previous studies, the alkoxy-phenols can re-polymerize toward char (thermal coke) but in the presence of the relatively strong Brønsted acidity of ZSM-5 zeolite they can undergo dehydration, decarbonylation, decarboxylation, dealkoxylation and cracking (C-C breaking) reactions, followed by enhanced formation of aromatics either via deoxygenation of the produced mono-phenolics or via aromatization of the small alkenes C2 = and C3 = formed initially via thermal or catalytic cracking or via dehydration of small intermediate alcohols (Mullen and Boateng, 2010; Ma et al., 2012; Yu et al., 2012; Ben and Ragauskas, 2013; Chu et al., 2013; Wang et al., 2014). All the above suggested pathways are depicted in the overall reaction mechanism shown in Figure 13.

The formation of ethylene and propylene has been verified in the present work by the use of ZSM-5 (Figure 11 and Table S4), thus supporting further the pathway of their aromatization toward mono-aromatics (Figure 13). These small alkenes may form via cracking of the C2/C3 side chains of the intermediate phenolics or via dehydration of intermediate small alcohols derived from the cleavage of the β-O-4 ether bonds (Ben and Ragauskas, 2013). The reactivity of ZSM-5 zeolite in the dealkoxylation of the primary produced alkoxy-phenols toward alkyl-phenols (as well as phenol via additional demethylation) was clearly shown by the results of the present work, both in the Py/GC-MS and in the fixed-bed reactor experiments. Interestingly, an inter-balance between aromatics and alkyl-phenols was observed, with the microporous ZSM-5(40) zeolite shifting the balance toward aromatics while the nano-sized and mainly the mesoporous ZSM-5 favoring the alkyl-phenols. Preliminary tests on the catalytic pyrolysis of model compounds (i.e., guaiacol and vanillin) has shown that they are mainly converted to alkyl-phenols (especially with the mesoporous ZSM-5) with few aromatics being also formed. It can thus be suggested that the prevailing route for aromatics formation is that via aromatization of small alkenes. Furthermore, it is also evident that the conventional microporous ZSM-5(40) zeolite with a crystal size of ~0.3–1 μm exhibit higher aromatization activity compared to the nano-sized and mesoporous ZSM-5 zeolites, thus proving the beneficial combination of strong Brønsted acidity with the appropriate space confinement offered by the ~5.5 A channels of the MFI structure. On the other hand, the high textural porosity of the nano-sized ZSM-5 zeolite favors the subsequent polymerization of the initially formed mono-aromatics, thus resulting in higher coke yields and slightly reduced aromatics. The mesoporous ZSM-5 zeolite with an average mesopore width of ~9 nm prepared by mild alkaline treatment, exhibits less polymerization and coking activity compared to nano-sized ZSM-5, being closer to the performance of conventional ZSM-5. The benefit however that is provided by the mesoporous ZSM-5 is the parallel high selectivity toward mono-aromatics and alkyl-phenols which can be attributed to the high mesopore volume and surface area that enhances the dealkoxylation of the relatively large alkoxy-phenols formed initially via thermal pyrolysis (Figure 13).

## Conclusions

The thermal (non-catalytic) and catalytic fast pyrolysis (CFP) of lignin has been studied with emphasis on the effect of process parameters and type of ZSM-5 zeolite catalyst on the selectivity of phenolics and aromatics in the bio-oil. By combining the detailed characterization of the softwood kraft lignin used by 2D HSQC NMR with the Py/GC-MS thermal pyrolysis tests, it was shown that the main composition profile of the lignin feedstock, in terms of phenylpropane units, is being “transferred” to the bio-oil. In this case, the thermal pyrolysis oil consisted mainly of guaiacol type compounds (i.e., alkoxy-phenols with single methoxy-group) originating from the abundant coniferyl (guaiacyl, G-units) alcohol units in the structure of the softwood (mainly spruce) kraft lignin. At lower pyrolysis temperatures, ca. 400°C, relatively larger guaiacol-type compounds were identified with C2/C3 alkyl side chains as well as oligomers consisting of two coniferyl alcohol units, while at higher temperatures (i.e., 500 and 600°C) smaller alkoxy-phenols, i.e., guaiacol, creosol and vanillin, prevailed. The production of char was also decreased by increasing the pyrolysis temperature.

In the catalytic fast pyrolysis experiments with the ZSM-5 zeolite, it was shown that the higher temperature (i.e., from 500 to 600°C) and the higher catalyst-to-lignin (C/L) ratio favored the conversion of the initially formed (due to thermal pyrolysis) alkoxy-phenols to mono-aromatics (benzene, toluene, p-xylene, o-xylene, 1,3-dimethyl-benzene, etc.), PAHs (mainly naphthalenes) and alkyl-phenols (phenol, 2-methyl-phenol, 2,3-dimethyl-phenol, etc.). With regard to the effect of the Si/Al of ZSM-5 zeolite (i.e., 11.5, 25, and 40), the proper combination of relatively high Si/Al (ca. 40) and C/L (ca. 4) in the pyrolysis tests (Py/GC-MS system) can induce higher conversion of alkoxy-phenols and higher selectivity toward mono-aromatics compared to alkyl-phenols. It was also shown that by increasing the intensity of the reaction conditions, i.e., higher temperature (500 vs. 600°C) and C/L (up to 4, in the Py/GC-MS system) the formation of solids (char plus coke on catalysts) were reduced.

When comparing the performance of three ZSM-5 zeolites with different characteristics, i.e., a commercial conventional microporous ZSM-5(40) zeolite, a synthesized nano-sized ZSM-5 (≤20 nm), and a mesoporous ZSM-5(9nm) prepared via alkaline treatment of the ZSM-5(40) zeolite (all having similar Si/Al ratio), it was shown that the well-crystallized ZSM-5(40) catalyst (with crystal size in the range of ca. 0.3–1 μm) exhibited slightly higher selectivity toward mono-aromatics compared to the other two catalysts. The nano-sized ZSM-5 and especially the mesoporous ZSM-5 showed in addition remarkably higher selectivity toward alkyl-phenols. However, both the mesoporous and mostly the nano-sized ZSM-5 induced the production of higher catalytic coke compared to the microporous ZSM-5 (from the Py/GC-MS tests). The evaluation of the same ZSM-5 zeolites in the fixed bed unit verified their performance with regard to their effect on bio-oil composition while it further showed that the total liquids (bio-oil), gas and coke yields by the mesoporous ZSM-5 were similar to those of the microporous ZSM-5. However, water production was higher with the meso-ZSM-5, thus leading to lower yield of organic bio-oil. On the other hand, the nano-sized ZSM-5 produced less total liquids with substantially less organic phase and significantly higher non-condensable gases and coke on catalyst. The observed differences in the performance of the microporous, mesoporous and nano-sized ZSM-5 zeolites were attributed to reaction pathways that may occur during lignin pyrolysis, comprising of initial thermal depolymerization reactions (C-O cleavage of the ether bonds in lignin), cracking of the side chains and dealkylation of the intermediate larger alkoxy-phenols toward the production of small (C2, C3) alkenes that may then be converted to mono-aromatics on the strong Brønsted acid sites of ZSM-5 zeolite channels, dealkoxylation (C-O cleavage) of the all the initially formed alkoxy-phenols toward the corresponding alkyl-phenols and phenol, and the less favored dehydrogenation/dehydration (C-O cleavage of the phenyl-OH bond) toward (alkyl)benzenes. The overall reaction mechanism scheme is complemented by polymerization/condensation reactions, either thermally or catalyzed by the acid sites of ZSM-5, of mono-aromatics and PAHs to reaction coke as well as of alkoxy-phenols to thermal char.

## Author contributions

PL conducted the experimental work (catalyst preparation and characterization, lignin characterization, pyrolysis tests, analysis of results) and drafted the manuscript. AF contributed in lignin analysis by NMR and in manuscript proof reading. SK contributed in catalysts preparation and characterization. KT organized and coordinated the research and experimental work and reviewed the final version of the manuscript.

### Conflict of interest statement

The authors declare that the research was conducted in the absence of any commercial or financial relationships that could be construed as a potential conflict of interest.
