# Supplementary material for: Catalytic Fast Pyrolysis of Kraft Lignin With Conventional, Mesoporous and Nanosized ZSM-5 Zeolite for the Production of Alkyl-Phenols and Aromatics
Source: Front Chem. 2018 Jul 18;6:295. doi: 10.3389/fchem.2018.00295 (PMC6058026; doi:10.3389/fchem.2018.00295)
Supplement: Supplementary file 1 [file Data_Sheet_1.PDF]

Supplementary Material

Catalytic fast pyrolysis of kraft lignin with conventional, mesoporous and nanosized ZSM-5 zeolite for the production of alkyl-phenols and aromatics

Polykarpos A. Lazaridis, Apostolos Fotopoulos, Stamatia Karakoulia and Konstantinos S. Triantafyllidis*

*** Corresponding author:** ktrianta@chem.auth.gr

Materials and Methods

Preparation of Meso-ZSM-5 (9 nm) by mild alkaline treatment

For the preparation of the mesoporous zeolite, named as Meso-ZSM-5 (9nm, des.), the commercial NH_4-ZSM-5 zeolite (CBV 8014, Si/Al = 40) was initially caclined at 500 ºC for 3 hours to obtain its proton form and was then subjected to mild alkaline treatment with a 0.2 M NaOH solution for 30 min at 65 ºC, under stirring. The spherical flask was then immersed into a cold bath to achieve instantaneous cooling, in order to control/stop the desilication of the zeolite, followed by filtration, washing of the zeolite with deionized water until pH~8 and drying overnight at 100ºC. The desilicated zeolite sample was then treated with 0.1 N HCl aqueous solution for 6 hrs at 65 ºC to remove the generated extra-framework Al species as well as the sodium ions, thus producing again the proton form of the desilicated ZSM-5 zeolite. The zeolite was then recovered by filtration, washing with deionized water until pH~6 and drying overnight at 100ºC.

Detailed description of Pyrolysis reactors (Py-GC/MS & Fixed bed reactor)

Pyrolysis tests using Py/GC-MS system

The thermal and catalytic fast pyrolysis experiments of kraft lignin were performed on a Multi-Shot Micro-Pyrolyzer (EGA/PY-3030D, Frontier Laboratories, Japan) connected to a gas chromatographer - mass spectrometer system (GCMS-QP2010, Shimadzu). The interface temperature between the micropyrolyzer and GC was set to 300°C and pyrolysis tests were conducted at 400-600 °C for 12 s. For the thermal fast pyrolysis experiments, a dried (80ºC under vacuum for 6 h) mixture of approximately 1 mg lignin and 2 mg silica sand (used as inert heat carrier) was loaded in a stainless-steel cup which was automatically lowered into the preheated furnace. In the case of the catalytic fast pyrolysis (CFP) experiments, a dried mixture of 1-4 mg calcined catalyst with 1 mg lignin (catalyst to lignin ratio range: 1 - 4) was loaded in the stainless steel cups for the respective pyrolysis experiments. The sample cups were weighed before and after pyrolysis by using a Mettler Toledo microbalance, with an accuracy of 0.00001 gr, to determine the initial sample weight and the weight of the residual char or char/coke. Helium (99.999%) was employed as the carrier gas at a flow rate of 100 ml min^{-1} in

the micropyrolyzer, with injector split ratio of 1:150 and 1 ml min^{-1} in the GC column. A capillary column was used (Ultra Alloy-5, Frontier Laboratories, Japan) with stationary phase consisting of 5% diphenyl and 95% dimethylpolysiloxane (30 m × 0.25 mm and 0.25 μm film thickness). The GC oven was programmed for a 4 minute hold at 40°C followed by heating (5°C min^{-1}) up to 300°C, where it was held constant for 7 minutes. The injector and detector temperature was kept at 300°C. The mass spectra were recorded in the range of m/z 45 to 500 with a scan speed of 5000 amu/s. Identification of mass spectra peaks was achieved by the use of the scientific library NIST11s. The derived compounds were classified and categorized in the following 16 groups-families: mono-aromatics (AR), aliphatics (ALI), phenols (PH), acids (AC), esters (EST), alcohols (AL), ethers (ETH), aldehydes (ALD), ketones (KET), polycyclic aromatic hydrocarbons (PAHs), sugars (SUG) nitrogen compounds (NIT), sulfur compounds (SUL), oxygenated aromatics (OxyAR), oxygenated phenols (OxyPH) and unidentified compounds (UN). To assess the precision of the experimental data, three replicate runs were performed for each experiment. A schematic representation of the Py/GC-MS system is shown in Supplementary Figure 1.

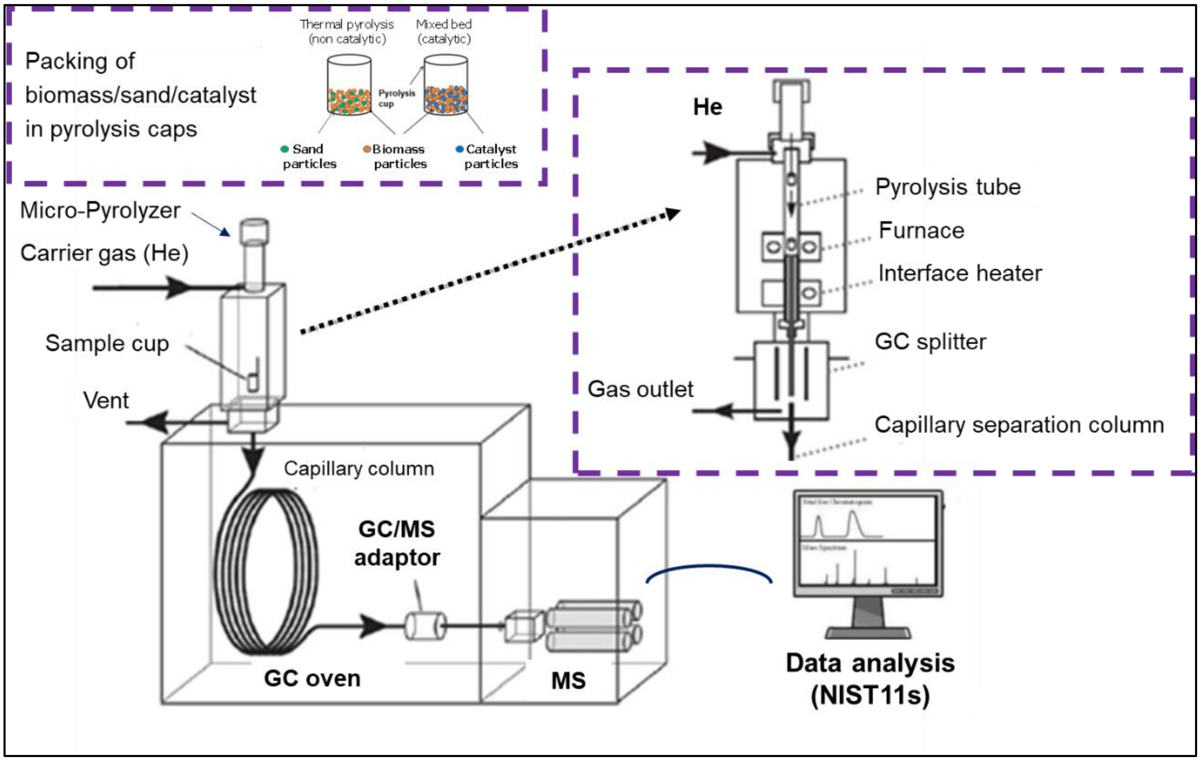

Figure S1. Schematic representation of Py/GC-MS system.

Pyrolysis tests on a bench-scale fixed bed reactor

Thermal and catalytic fast pyrolysis tests of kraft lignin were also performed on a bench-scale fixed bed tubular reactor (ID 1.02 cm, height 35.5 cm), made of stainless steel 316 and heated by a 3-zone furnace. A specially designed piston system was used to introduce the solid lignin into the reactor. The amount of lignin (dried at 80°C under vacuum for 6 h) used was typically 0.5 g and the amount of silica sand (in the non-catalytic, thermal pyrolysis experiments) or catalyst (in the catalytic experiments) was also 0.5 g. A constant stream of N_2 (100 cc/min) was fed from the top of the reactor during the pyrolysis experiments for the maintenance of the inert atmosphere during pyrolysis and the continuous withdrawal of the product vapors. In a typical experiment, lignin was inserted from the top of the reactor instantaneously with the aid of the piston in the preheated reactor zone and was initially

pyrolyzed/vaporized at 600 ^oC on a hot quartz-wool layer placed on the top of the catalyst bed. The produced pyrolysis vapours were then driven downwards through the catalyst's bed with the aid of the constant N_2 flow (100 cm^3/min) for 20 min. The pyrolysis product vapors were condensed in pre-weighted spiral glass receivers placed in a cooling bath (dry ice, -78.5°C). The obtained bio-oil was collected with absolute ethanol as solvent and homogenized before the analysis by GC–MS (GCMS-QP2010, Shimadzu). The NIST11s mass spectral library was used for the identification of the compounds in the bio-oil, while internal libraries and software were used for their categorization into main functional groups-families, as in the case of Py/GC-MS experiments. The water content of bio-oil was determined by Karl-Fischer titration (ASTM E203-08), while the elemental analysis (C/H/N/S) of the organic fraction of the bio-oil was determined by LECO 628 and LECO 932 analyzers (USA); O was determined by difference. The non-condensable gases (NCG's) products were collected and analyzed in a HP 6890 GC, equipped with four columns (Precolumn:OV-101; Columns: Porapak N, Molecular Sieve 5A and Rt-Qplot (30m×0.53mm i.d.) and two detectors (TCD and FID). The amount of solids, which comprised of char and sand in the non-catalytic pyrolysis experiment, char plus coke-on-catalyst and catalyst in the catalytic pyrolysis experiments, as well as the quartz wool used to separate the two bed-zones, was determined by direct weighing. An indirect estimation of the coke formed on the catalyst, as wt.% on initial lignin, was performed by subtracting the measured char content of the non-catalytic experiment from the char+coke content of the catalytic experiments (char formation is not affected by the presence of the catalysts, as lignin and catalysts do not come in contact, see Fig. S2 and related description above). Furthermore, the decomposition profile of the collected char and coke (on the spent catalysts) was studied by thermogravimetric analysis (TGA, NETZSCH STA 449 F5 Jupiter) using dry air as carrier gas, at a flow rate of 50 mL/min. The samples were heated from room temperature to 850 °C at heating rate of 10oC/min. A schematic representation of the bench-scale fixed bed tubular reactor for lignin pyrolysis is shown in Supplementary Figure 2.

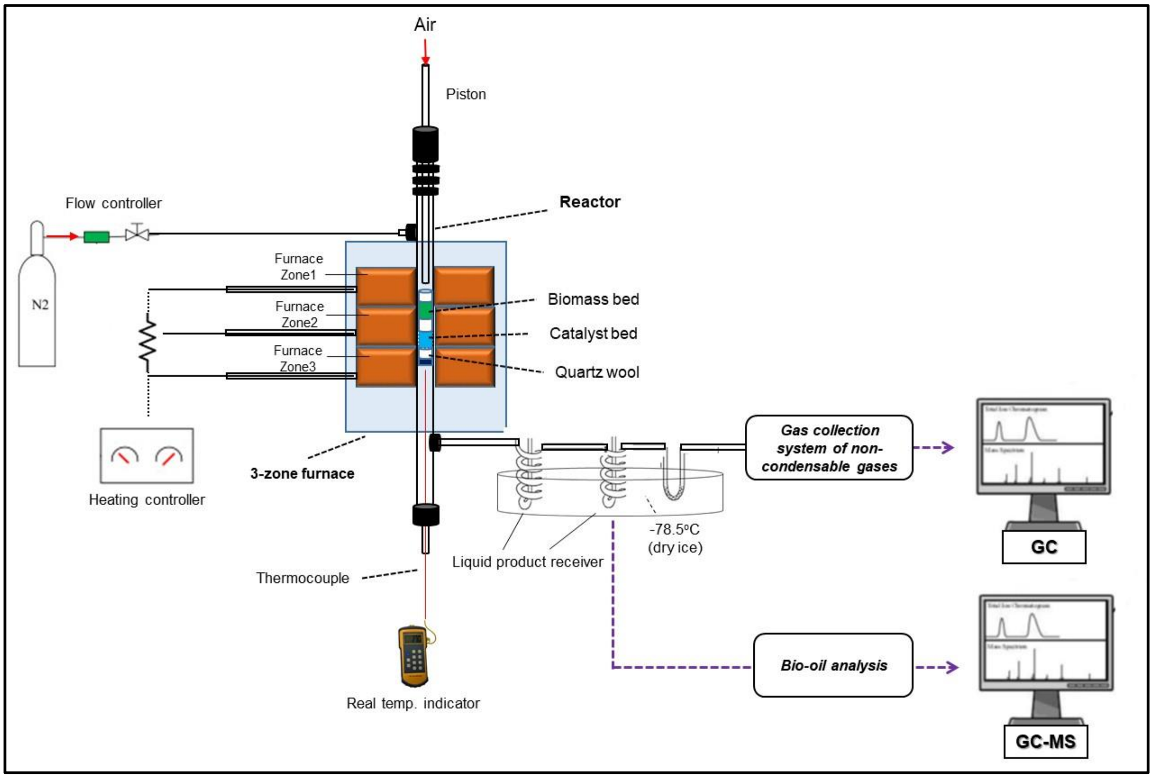

Figure S2. Schematic representation of bench-scale fixed bed tubular reactor for lignin pyrolysis.

Results & Discussion

Analysis of kraft lignin by 2D HSQC NMR

Table S1. Assignment of kraft lignin ^{13}C - ^{1}H cross-signals in the HSQC spectra.

Label	δ_C/δ_H (ppm)	Assignment
B_β	53.5/3.1	C_β-H_β in resinol substructures (B)
A_γ	60.0/3.4	C_γ-H_γ in β-O-4' substructures (A)
C_γ	60 and 60.3/3.8	C_γ-H_γ in phenylcoumaran substructures (C)
I_γ	61.5/4.1	C_γ–H_γ in cinnamyl alcohol end-groups (I)
B_γ	70.9/4.16	C_γ-H_γ in resinol substructures (B)
A_α	71/4.8	C_α-H_α in β-O-4′ substructures (A)
A_β	84.1/4.3	C_β-H_β in β-O-4' substructures (A)
B_α	84.9/4.6	C_α-H_α in β-β resinol (B)
C_α	86.8/5.5	C_α-H_α in phenylcoumaran substructures (C)
G_2	109.5-112.8/6.7-7.2	C_2-H_2 in guaiacyl units (G)
$G_{2'}$	110.6 and 111.1/7.4	C_2-H_2 in oxidized (Cα=O) guaiacyl units (G')
G_5	115.3/6.8	C_5-H_5 in guaiacyl units (G)
G_6	118.7-121.8/6.5-7.3	C_6-H_6 in guaiacyl units (G)
J_β	125.6/7.0	C_β-H_β in cinnamaldehyde end groups (J)
$G_{6'}$	126/7.5	C_6-H_6 in oxidized (Cα=O) guaiacyl units (G')

Catalysts characterization

Determination of porosity characteristics (Ar and N_2 physisorption)

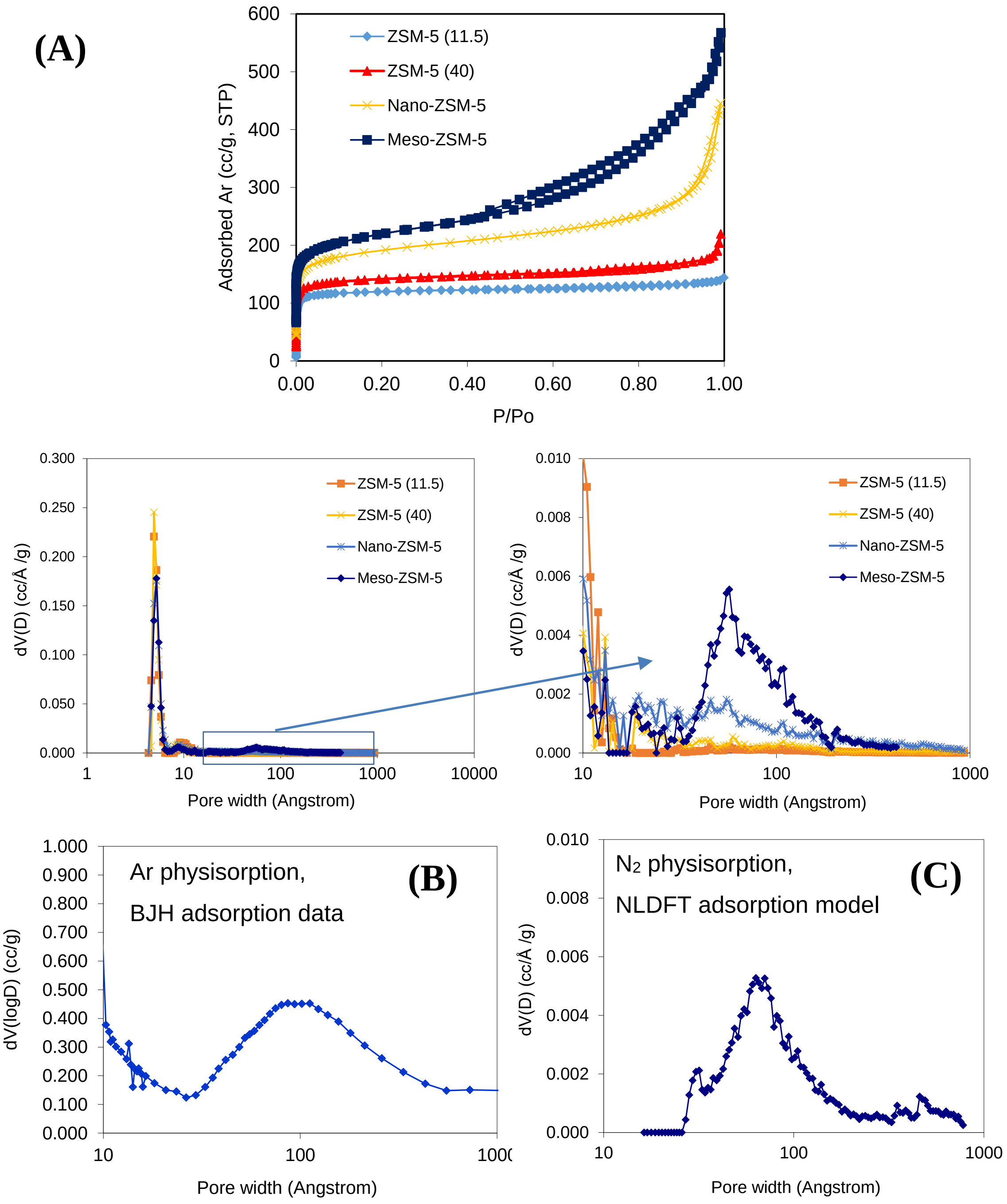

Figure S3. (A) Ar adsorption-desorption isotherms and NLDFT pore width distribution curves of the conventional microporous ZSM-5 (Si/Al = 11.5, 40) zeolites, the nanosized ZSM-5 zeolite synthesized under controlled/mild template hydrothermal synthesis, and the mesoporous ZSM-5 zeolite prepared by mild alkaline treatment of the conventional ZSM-5(40) zeolite. **(B)** BJH pore width distribution curve from Ar physisorption of the mesoporous ZSM-5. **(C)** NLDFT pore width distribution curve from N_2 physisorption of the mesoporous ZSM-5.

Table S2. Porosity characteristics derived from Ar physisorption measurements at -186°C

Catalyst	BET area[a] (m^2/g)	Micro-pore area[b] (m^2/g)	Meso/ macropore & external area[c] (m^2/g)	Average mesopore width (nm) (BJH, adsorption data)	Average mesopore width (nm) (NLDFT adsorption model)
ZSM-5(11.5)	410	363	47	-	-
ZSM-5(40)	464	385	79	-	-
Meso-ZSM-5 (9nm)	507	235	272	~9.0	~5.7
Nano-ZSM-5	489	274	215[d]	-	-

[a] BET area from Ar sorption at -186°C, Multi-point BET method; [b] t-plot method; [c] Difference of BET area minus micropore area; [d] Attributed mainly to macropores and external surface area

Determination of acidic properties of the zeolites by FT-IR coupled with in situ pyridine adsorption

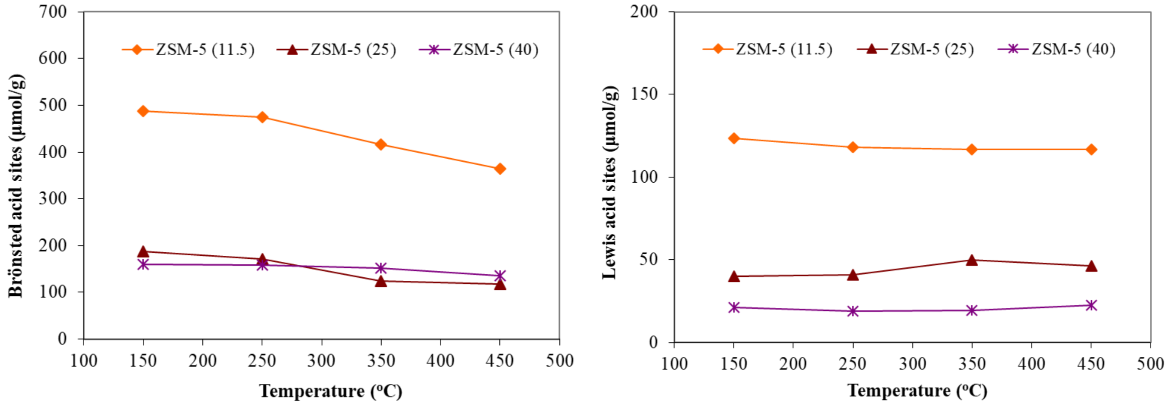

Figure S4. Content of Brønsted and Lewis acid sites of the microporous ZSM-5 zeolitic catalysts with different Si/Al ratio, as determined by FTIR measurements of in situ sorbed pyridine at increasing equilibration temperature. A greater number of acid sites (i.e., higher amount of sorbed pyridine) retained at higher temperatures indicates higher relative acid strength of the catalyst.

Py/GC-MS lignin pyrolysis results

Table S3. Composition of bio-oil derived from non and catalytic fast pyrolysis of kraft (spruce) lignin in the Py/GC-MS system (GC-MS peak area, %). The catalytic results refer to experiments at 600ºC with catalyst to lignin (C/L) ratio of 4

Categories/ Compounds	Thermal (on-catalytic) pyrolysis			Catalytic pyrolysis (600ºC, catalyst to lignin (C/L) = 4 w/w		
	400ºC	500ºC	600ºC	ZSM-5 (40)	Nano-ZSM-5	Meso-ZSM-5 (9nm)
	GC-MS Peak Area (%)					
**Aromatics (AR)**						
Toluene		0.06	0.1	13.14	12.53	8.96
Benzene				3.63	1.85	2.31
p-xylene				3.46	10.49	
o-xylene				4.19		
Benzene, 1,3-dimethyl-				6.30	0.74	11.88
Benzene, 1,2,4-trimethyl-					1.29	3.13
Benzene, 1-ethyl-2-methyl-						
Benzene, 1,2,3-trimethyl-						
Ethylbenzene						
Indane						0.72
Indene				3.10		1.41
1H-Indene, 1-methyl-				3.23		1.96
1H-Indene, 1-methylene-					2.34	
**Aliphatics (ALI)**						
Azulene				6.08		2.57
Hexadecane						1.01
**Phenols (PH)**						
Phenol		0.81	0.81	2.66	2.08	4.47
p-Cresol		1	1.25			
Phenol, 4,4'-(1-methylethylidene)bis-		1.48				
Phenol, 2-methyl-		0.16	1.21	7.48	4.40	6.56
Phenol, 3-methyl-	0.06	0.78			6.34	9.22
Phenol, 2,3-dimethyl-			0.48	2.60		2.75
Phenol, 2,4-dimethyl-			1.16		4.82	
Phenol, 2,5-dimethyl-					1.88	6.33
Phenol, 2,6-dimethyl-				1.68	1.49	1.00
Phenol, 2,3,6-trimethyl-						
Catechol			0.81			
**Furans (FUR)**						
Benzofuran, 2,3-dihydro-	0.14		0.44			
5-Isopropyl-3,3-dimethyl-2-methylene-2,3-dihydrofuran	1.05	0.81	0.18			
Benzofuran, 2-methyl-				2.29	3.71	2.13
**Acids (AC)**						
**Esters (EST)**						
7-Oxodehydroabietic acid, methyl ester	0.19					
1,3-Benzenedicarboxylic acid, bis(2-ethylhexyl) ester	0.06					

**Alcohols (AL)**						
**Ethers (ETH)**						
**Aldehydes (ALD)**						
3-Ethoxy-4-methoxybenzaldehyde			0.29			
**Ketones (KET)**						
7-(3,4-Methylenedioxy)-tetrahydrobenzofuranone	5.64	3.21	3.06			
Dibenz[d,f]cycloheptanone, 2,3,9-trimethoxy-	0.72	0.48	0.40			
**Polycylic aromatic hydrocarbons (PAHs)**						
Naphthalene				1.35	2.78	0.66
Naphthalene, 1-methyl-				8.25	6.50	5.53
Naphthalene, 2-methyl-				2.34	2.94	
Naphthalene, 1,7-dimethyl-					2.00	0.23
Naphthalene, 2,6-dimethyl-				4.18		2.26
Anthracene					1.19	0.43
**Sugars (SUG)**						
**Nitrogen compounds (NIT)**						
3-(3-Hydroxy-4-methoxyphenyl)-l-alanine	1.51	1.23				
**Sulfur compounds (SUL)**						
**Oxygenated aromatics (OxyAR)**						
3,4-Dimethoxytoluene	0.72	0.83	0.38		0.19	0.25
Benzene, 4-ethenyl-1,2-dimethoxy-	0.13	0.45	0.39		0.40	
1,2-Propanediol, 3-benzyloxy-1,2-diacetyl-						
**Oxygenated phenolics (OxyPH)**						
Phenol, 2-methoxy-	17.4	14.48	15.16	1.00	3.20	0.90
2-Methoxy-5-methylphenol	0.64	0.31	0.40	1.15		0.51
Creosol	21.98	20.38	18.84	2.46	7.85	1.98
Phenol, 4-ethyl-2-methoxy-	6.78	7.43	7.06	0.57	2.09	
2-Methoxy-4-vinylphenol	11.94	8.13	3.17		0.59	0.23
Phenol, 2-methoxy-5-(1-propenyl)-, (E)-	1.13					
Phenol, 2-methoxy-4-propyl-	1.13	1.13	1.01		0.29	
Phenol, 2-methoxy-4-(1-propenyl)-	7.45	1.94	1.73			
Phenol, 2-methoxy-3-methyl-		0.76				0.27
Eugenol		1.35	1.63			
Trans-isoeugenol		1.74	3.92			
Vanillin	1.18	4.90	6.48			
Apocynin		2.24	2.57			
2-Propanone, 1-(4-hydroxy-3-methoxyphenyl)-	0.78	1.75	1.64			
Phenol, 4,4'-(1-methylethylidene)bis-		1.48				
Phenol, 2-methoxy-4-(1-propenyl)-, (Z)-		1.70				
Ethyl homovanillate	1.28		0.91			
4-Hydroxy-3-methylacetophenone			6.13			
Carinol	7.64	5.14	3.14			
(-)-Nortrachelogenin	0.71	2.44	0.79			

Table S4. Char/coke formation after thermal (non-) and catalytic fast pyrolysis of kraft lignin in the Py/GC-MS system.

Feed biomass	Catalyst	Conditions		Char/coke (wt. %)
		Pyrolysis temp. (ºC)	C/L ratio	
**Thermal fast pyrolysis**				
Kraft	Silica sand	400	2	54.4
Kraft	Silica sand	500	2	43.3
Kraft	Silica sand	600	2	34.5
**Catalytic fast pyrolysis using micro- and nanosized zeolites**				
Kraft	ZSM-5 (11.5)	600	2	32.4
Kraft	ZSM-5 (11.5)	600	4	30.6
Kraft	ZSM-5 (25)	600	2	31.5
Kraft	ZSM-5 (25)	600	4	29.9
Kraft	ZSM-5 (40)	500	2	44.6
Kraft	ZSM-5 (40)	500	4	39.9
Kraft	ZSM-5 (40)	600	1	37.4
Kraft	ZSM-5 (40)	600	2	35.2
Kraft	ZSM-5 (40)	600	3	30.6
Kraft	ZSM-5 (40)	600	4	28.1
Kraft	Nano-ZSM-5	600	4	33.4
Kraft	Meso-ZSM-5 (9 nm)	600	4	30.3

Fixed-bed lignin pyrolysis results

Table S5. Product yields (wt.% on lignin) and bio-oil composition from the thermal and catalytic pyrolysis of kraft lignin on fixed-bed reactor at 600◦C with various ZSM-5 zeolites (C/L=1).

	Kraft + sand (non catalytic)	Kraft + ZSM-5(40)	Kraft + Nano-ZSM-5	Kraft + Meso-ZSM-5 (9 nm)
**Total liquids**	36.6	25.6	20.3	24.9
Organic bio-oil	30.6	16.2	7.6	12.1
Water	6.1	9.3	12.7	12.8
**Total gases**	14.0	22.0	26.4	23.6
**Solids (char + coke on catalyst)**	42.2	47.9	49.9	48
**Coke on catalyst** (difference of char+coke minus char from the non-catalytic experiment)	-	5.7	7.7	5.8
**Elemental composition of the organic bio-oil (wt.% on organic bio-oil)**				
Carbon	66.8	79.7	76.5	77.1
Hydrogen	7.5	9.2	9.9	9.8
Oxygen	25.0	10.5	12.8	12.6
Sulfur	0.07	0.05	0.06	0.05

**Non-condensable gases (wt.% on lignin)**				
CO_2	6.4	8.9	9.8	8.9
CO	4.1	8.0	8.9	8.5
CH_4	3.2	3.6	4.6	3.1
Ethane	-	0.3	0.4	0.4
Ethylene	-	1.4	1.6	1.6
Propane	0.3	0.2	0.2	0.3
Propylene	-	0.6	0.9	0.8
**GC–MS analysis (Bio-oil composition, % GC-MS peak area)**				
Aromatics	0.10	32.46	26.57	25.12
Aliphatics	0.82	0.12	0.00	0.00
Phenols	5.65	32.33	42.85	45.91
Furans	0.00	1.94	2.46	0.44
Acids	3.01	0.00	0.00	0.00
Esteres	0.36	0.00	0.00	0.00
Alcohols	0.00	0.00	0.64	0.00
Etheres	0.00	0.27	0.32	0.00
Aldehydes	0.32	0.13	0.00	0.00
Ketones	5.14	0.15	0.00	0.00
PAH’s	0.95	30.76	22.24	25.01
Sugars	0.00	0.00	0.00	0.00
Nitrogen compounds	1.36	0.12	0.00	0.00
Sulfur compounds	0.00	0.09	0.00	0.00
Oxygenated aromatics	4.10	1.33	0.35	0.00
Oxygenated phenols	75.42	0.31	4.35	0.00
Unidentified	2.78	0.00	0.22	0.00

Thermal decomposition (in air) of char and coke-on-catalyst

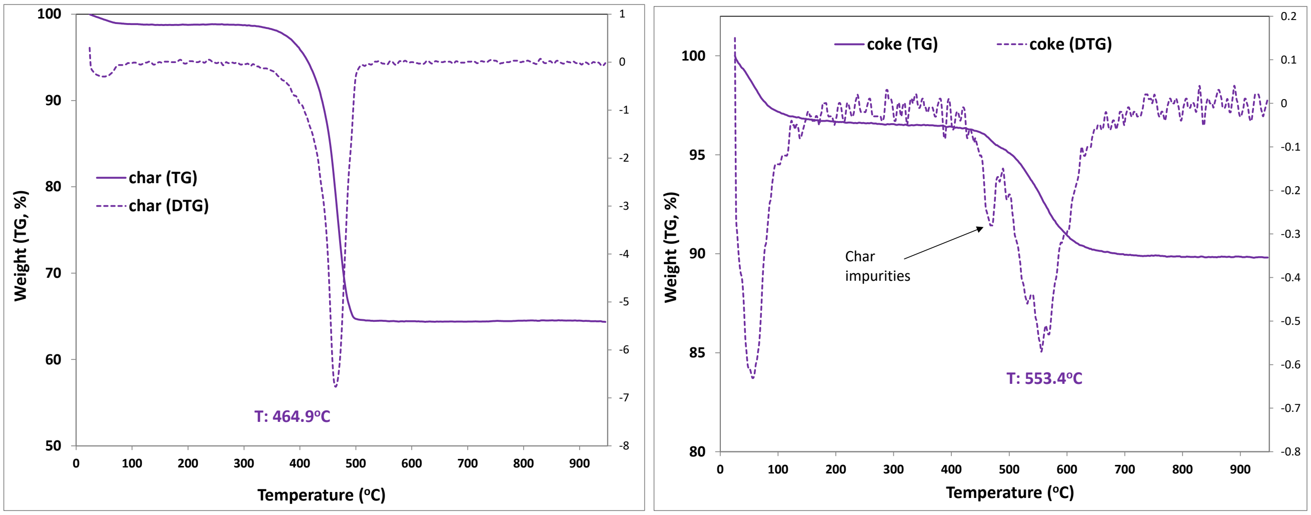

Figure S5. TGA/DTG curves of char and coked catalyst (nano-ZSM-5 zeolite) after lignin pyrolysis in the fixed bed reactor (10ºC/min, air flow, 50 cc/min). The “char impurities” refer to small amounts of char from the lignin thermal pyrolysis zone (upper zone in the fixed bed reactor, Fig. S2) which hasn’t been completely separated from the coked catalyst zone (lower zone in the reactor) after the experiment.

Table S6. Sulfur (wt.%) content in the char or coked catalyst that were collected from the fixed bed reactor after kraft lignin pyrolysis experiments at 600◦C with various ZSM-5 zeolites (C/L=1).

	Char (non catalytic)	Kraft + ZSM-5(40) char	Kraft + ZSM-5(40) coke	Kraft + Nano-ZSM-5 char	Kraft + Nano-ZSM-5 coke	Kraft + Meso-ZSM-5 (9 nm) char	Kraft + Meso-ZSM-5 (9 nm) coke
**S (wt.%)**	-	0.552	-	0.333	0.359	0.335	0.159
